# Research Progress on Irradiation Damage in High-Performance Nickel-Based Alloys for Advanced Reactors

**DOI:** 10.3390/nano16191214

**Published:** 2026-09-25

**Authors:** Heding Meng, Guanyu Liu, Tianyi Hu, Feida Chen, Longjingrui Ma, Qing Peng, Bin Cai, Hai Huang

**Affiliations:** 1 Key Laboratory of Material Physics, Ministry of Education, School of Physics, Zhengzhou University, Zhengzhou 450001, China; 2 Department of Nuclear Science & Technology, Nanjing University of Aeronautics and Astronautics, Nanjing 210016, China; 3 National Key Laboratory of Thorium Energy, Shanghai Institute of Applied Physics, Chinese Academy of Sciences, Shanghai 201800, China; 4 School of Power and Mechanical Engineering, Wuhan University, Wuhan 430072, China

**Keywords:** nickel-based alloys, advanced nuclear reactors, irradiation damage, irradiation-resistance strategies

## Abstract

Gen-IV nuclear systems demand structural materials with superior high-temperature mechanical integrity, corrosion resistance, and irradiation tolerance. Nickel-based alloys, owing to their excellent high-temperature properties, chemical stability, and irradiation resistance, have become leading candidate materials for key components in advanced reactors such as molten salt reactors and very-high-temperature gas-cooled reactors. This review comprehensively surveys typical reactor-grade nickel-based alloys—Hastelloy N/GH3535, Inconel 718, and Inconel 617—covering their in-service behavior, key properties, processing–microstructure relationships, and irradiation damage research. Special attention is given to irradiation-induced microstructural evolution, including point defect generation and migration during cascades, formation of dislocation loops and voids, helium bubble nucleation and growth, precipitation, and phase transformations, as well as their links to macroscopic property degradation (hardening, embrittlement, swelling, and creep). Recent advances in ion-irradiation experiments, microstructural characterization, nanomechanical testing, and computational methods (first-principles and molecular dynamics) are critically discussed. It is shown that irradiation resistance can be significantly enhanced through compositional optimization, grain-boundary and dislocation engineering, oxide-dispersion strengthening, and the design of heterogeneous interfaces. Finally, future research directions are proposed, emphasizing advanced characterization, multiscale modeling, and data-driven approaches for accelerating the development of next-generation irradiation-resistant nickel-based alloys.

## 1. Introduction

Against the backdrop of addressing global warming, energy depletion, and environmental pollution, nuclear energy has emerged as a primary alternative to fossil fuels for building a modern clean energy system, owing to its high energy density, zero carbon emissions, and inherent stability [1]. The nuclear reactor, as the core device for controlled fission, underpins the safe and efficient utilization of nuclear energy, and its technological evolution is now marked by advancing Gen-IV concepts—such as the gas-cooled fast reactor (GFR), very-high-temperature reactor (VHTR), and molten salt reactor (MSR)—which, compared to conventional light water reactors, feature significantly higher operating temperatures and neutron irradiation damage doses, thereby imposing more stringent demands on the comprehensive performance of key structural materials in extreme service environments; a comparative overview of these environmental demands for various advanced nuclear systems is presented in Figure 1 [2].

The microstructure and property evolution of nuclear reactor structural materials is intrinsically tied to their service environment: from the aluminum and magnesium alloys selected for early low-temperature, low-power-density natural-uranium reactors owing to their low neutron absorption cross-sections, to the austenitic stainless steels and ferritic/martensitic (F/M) steels that later became mainstream for fast reactors, the development of structural materials has always been driven by the need to meet demanding in-reactor performance requirements [3,4]. However, during long-term operation, these materials are subjected to the synergistic effects of high temperature, high stress, aggressive coolants, and high-dose, high-energy neutron irradiation, along with transmutation products such as helium and hydrogen, leading to significant property degradation [5]. Extensive research has demonstrated that traditional structural materials, including austenitic stainless steels and F/M steels, are susceptible to severe irradiation damage—such as irradiation-induced phase transformations, voids, and dislocation loops—that impairs their mechanical and corrosion properties and induces irradiation-assisted stress corrosion cracking (IASCC), rendering them unable to fully withstand the extreme service conditions of Gen-IV nuclear reactors and thus posing a critical threat to operational safety [6,7,8,9].

In recent years, high-performance nickel-based alloys have emerged as prime candidate structural materials for core components of Gen-IV reactors (e.g., MSR, GFR, and VHTR) due to their outstanding performance in high-temperature, high-irradiation, and corrosive environments, and are widely used in the fabrication of critical parts for various advanced nuclear systems [10,11]. These alloys are based on a face-centered cubic (FCC) nickel matrix and achieve exceptional high-temperature strength, creep resistance, oxidation/corrosion resistance, and coolant compatibility through synergistic solid-solution and precipitation strengthening imparted by multi-component alloying with elements such as Cr, Mo, and Fe, thereby exhibiting comprehensive service performance significantly superior to that of conventional structural materials like austenitic stainless steels and F/M steels [12,13,14,15,16]. As a result, nickel-based alloys are extensively employed in key components such as reactor internals, heat exchangers, steam generators, and coolant pumps, where their property stability directly dictates the operational efficiency, service life, and safety of the entire nuclear energy system [17].

In light of the performance limitations of conventional structural materials in extreme irradiation environments, nickel-based alloys, by virtue of their substantial property advantages, have emerged as the leading candidate materials for critical components of Gen-IV reactors. However, they are still subject to significant irradiation damage under these service conditions, which critically undermines their long-term operational stability. To address this core challenge, this review systematically presents the latest research progress on irradiation damage in nickel-based alloys, focusing on representative alloy systems, damage mechanisms, experimental and computational methodologies, and strategies for enhancing irradiation resistance, with the aim of providing feasible pathways and insights for the design and development of irradiation-tolerant nickel-based structural materials for future advanced reactors. It should be emphasized that the selection of nickel-based alloys for Gen-IV reactor components is driven by their unique combination of high-temperature strength, molten salt corrosion resistance, and microstructural stability, which cannot be readily achieved with conventional steels or other candidate materials. Nevertheless, these alloys are known to suffer from severe irradiation-induced degradation, particularly transmutation helium embrittlement and void swelling, under the high neutron doses and elevated temperatures typical of advanced reactor concepts. Therefore, the present review does not advocate their unqualified use, but rather seeks to elucidate the underlying damage mechanisms and to critically assess current strategies—such as compositional optimization, microstructural engineering, and composite material design—that may mitigate these irradiation effects and extend the service life of nickel-based alloys in advanced nuclear systems.

## 2. Typical Nickel-Based Alloys for Advanced Reactors

Currently, the principal nickel-based alloy systems intensively investigated and deployed for advanced nuclear reactors are the Inconel (Ni–Cr and Ni–Fe–Cr) and Hastelloy (Ni–Cr–Mo) families, whereas the Monel (Ni–Cu) series is typically reserved for non-structural components due to its comparatively lower elevated-temperature strength and irradiation tolerance. This section focuses on three representative high-performance alloys—Hastelloy N, Inconel 718, and Inconel 617—presenting their current research status, typical application scenarios and service conditions, key performance metrics (including high-temperature mechanical properties, corrosion resistance, and irradiation tolerance), the effects of processing routes on microstructure and properties, as well as existing challenges and future development directions in advanced nuclear reactors.

### 2.1. In-Reactor Service of Typical Alloys

Hastelloy N alloy is a solid-solution-strengthened Ni–Mo–Cr nickel-based superalloy specifically developed for MSRs (designated GH3535 in China), with its chemical composition detailed in Table 1 [18]; owing to its excellent irradiation resistance, high-temperature strength, and corrosion resistance, it has been recommended as the primary structural material for MSRs. As the only liquid-fuel reactor concept among Gen-IV systems, the MSR employs molten fluoride salts as both coolant and fuel carrier, thereby exposing structural materials to a synergistic extreme environment of high-energy neutron irradiation and high-temperature fluoride salt corrosion that readily induces creep deformation along with microstructural evolutions such as void formation, dislocation loops, and precipitation, ultimately compromising reactor safety and service life [19,20,21,22]. Figure 2 shows the overall layout of the MSR Experiment (MSRE) at Oak Ridge National Laboratory, which accumulated over 13,000 h of full-power operation, during which Hastelloy N was extensively utilized in all major components [23,24]. Research on the alloy’s service behavior in high-temperature molten salts has demonstrated that subtle compositional modifications can significantly influence performance; for instance, Hu et al. [25] found that adding Li to molten FLiNaK salt markedly suppressed the interaction between GH3535 alloy and SiC, reducing the total corrosion and deposition layer depth from 200 μm to 30 μm after 1000 h. Regarding the irradiation–corrosion synergy, Ji et al. [26] compared the microstructures of GH3535 after Au-ion and He-ion irradiation, revealing that helium bubbles act as nucleation sites for Cr_2_O_3_, promoting its dispersed precipitation and accelerating outward Cr diffusion, which in turn exacerbates corrosion. Furthermore, our own group has identified several critical irradiation-induced phenomena in GH3535 under high-dose Au-ion irradiation, including lattice distortion and new phase formation, non-monotonic evolution of surface morphology, and irradiation-accelerated C atom clustering [27]. Consequently, the evolution of these irradiation-induced microstructural defects and their impact on chemical stability have become a vital basis for evaluating the service lifetime of the GH3535 alloy.

Inconel 718 is a precipitation-hardened Ni–Fe–Cr alloy containing Nb and Mo (composition detailed in Table 2 [28]), strengthened by γ′ (Ni_3_(Al,Ti)) and γ″ (Ni_3_Nb) precipitates in an austenitic γ matrix, and retains good microstructural and property stability over a wide temperature range from −253 °C to 650 °C; its high Cr content provides adequate corrosion resistance, and it also offers outstanding weldability, which is rare among nickel-based alloys [29]. Owing to these characteristics, Inconel 718 is extensively employed in pressurized water reactor fuel assembly components, such as grid springs and bolts [30]. During service, these critical components are subjected to multiple synergistic environmental attacks, including neutron irradiation-induced displacement damage, stress corrosion cracking, corrosion fatigue, crevice corrosion, pitting, and fretting wear, which collectively pose a severe threat to the long-term operational safety of nuclear facilities and significantly constrain the performance improvement and service life of fuel assemblies [31,32].

Inconel 617 is a solid-solution-strengthened Ni–Cr–Co–Mo superalloy developed by Special Metals Corporation, with its nominal composition given in Table 3 [33]; its high-temperature strength is primarily derived from solid-solution hardening by Co and Mo and precipitation strengthening from Cr- and Mo-rich carbides, while minor γ′ (Ni_3_(Al,Ti)) precipitates may provide auxiliary strengthening. The alloy exhibits excellent high-temperature oxidation and corrosion resistance owing to the formation of protective Cr_2_O_3_ and Al_2_O_3_ scales, which effectively suppress pitting and oxidation attack. Consequently, Inconel 617 is one of only six alloys approved by the ASME Code for elevated-temperature nuclear components and has become a leading candidate material for intermediate heat exchanger piping in VHTRs [34,35]. In VHTR service, the helium coolant inevitably contains low-level impurities such as H_2_, CH_4_, and H_2_O, which, at high temperatures, can aggressively react with the alloy, causing internal oxidation, carburization/decarburization, and deep corrosive degradation; furthermore, when combined with neutron irradiation, this corrosion–irradiation synergy can significantly accelerate material property deterioration, presenting a critical challenge to the long-term integrity of core components.

### 2.2. Key Performance Metrics

Over the past few decades, researchers worldwide have conducted extensive investigations—encompassing experimental characterization, theoretical modeling, and simulations—on the key performance evolution of nickel-based alloys under extreme reactor conditions, with the research focus progressively shifting from isolated mechanical property assessments to the analysis of complex synergistic effects among temperature, irradiation, and corrosion.

In the field of high-temperature mechanical behavior and modification, fatigue properties—particularly high-temperature low-cycle fatigue and creep–fatigue interaction—are of central importance as they govern the service life of reactor internals and have thus garnered sustained attention. To enhance fatigue resistance, advanced surface strengthening techniques have proven effective; for example, Wang et al. [36] demonstrated that laser shock peening (LSP) significantly improved the microhardness, residual stress distribution, microstructure, and fatigue performance of laser-clad repaired Inconel 718 alloy. In parallel with process modification, reliable fatigue life prediction is equally critical for safety assessment, and Qu et al. [37] computed and analyzed the fatigue data of age-hardened X-750 alloy using the Manson–Coffin relationship and the Zheng constitutive model—the latter being a strain–life parabolic relationship that introduces a critical strain fatigue limit (ε_0_) to account for the material’s fatigue limit, thereby overcoming the inability of the Manson–Coffin equation to reflect either the stress or strain fatigue limit and providing improved physical consistency with experimental observations. Their results showed that the Zheng model provides higher prediction accuracy from room temperature (RT) to 650 °C, thereby offering valuable experimental data and a computational basis for subsequent fatigue life prediction of nickel-based alloys.

Research on irradiation-induced microstructural and property degradation has focused on the evolution of defect damage under various ion irradiation doses and temperatures. For example, Huang et al. [38] reported that with increasing Ni-ion dose, the average size of irradiation-induced defects (such as solute clusters and dislocation loops) in GH3535 alloy remained essentially unchanged while their number density continuously increased. In contrast, when transmutation helium is present, more severe damage features can arise; Zhang et al. [39] compared Kr^2+^ ion and neutron irradiation in Inconel X-750 and found that no voids formed under Kr^2+^ irradiation up to 5.4 dpa at temperatures of 60–400 °C, whereas voids appeared after neutron irradiation to similar doses and temperatures, indicating that void formation is intimately linked to concurrent helium generation during neutron irradiation. The detrimental role of helium has been further elucidated by Njifon et al. [40], who demonstrated that helium production significantly increases the number of Frenkel pairs once a critical dose threshold is exceeded, with this threshold depending on the helium generation rate, thereby accelerating overall defect accumulation and property deterioration.

Research on high-temperature oxidation mechanisms and passivation has centered on the influence of temperature, exposure time, and cycling conditions on oxide scale evolution. Xu et al. [41] investigated the oxidation behavior of GH4738 under isothermal and non-isothermal conditions and found that excessively high temperatures induce substantial porosity at the interface between the inner oxide layer (IOL) and the internal oxidation zone (IOZ), while prolonged oxidation leads to coarsening of Al-rich oxide particles as the IOZ deepens. In a parallel study on GH4169, Yin et al. [42] reported that Fe-rich spinel oxides—notably (Fe,Cr)_3_O_4_—preferentially nucleate at grain boundaries with a distinctive spinel morphology, whereas Cr_2_O_3_ predominantly forms as granular particles within the grains; with increasing oxidation time, these oxide particles gradually grow and eventually interconnect into a dense, protective scale that isolates the underlying alloy from the environment and effectively blocks further degradation. These findings on oxide scale integrity and evolution provide critical baseline knowledge for evaluating the synergistic effects of oxidation and irradiation on the service stability of nickel-based alloys in advanced reactors.

Regarding alloy composition optimization and elemental influence, the addition of beneficial minor elements (e.g., Y) and the substitution of major alloying constituents (e.g., replacing Mo with W) can markedly enhance the irradiation resistance of nickel-based alloys. Liu et al. [43] demonstrated that an appropriate Y doping significantly suppresses irradiation hardening in Ni–Mo–Cr alloys, while Wei et al. [44] found that, relative to the Ni–Mo–Cr system, a Ni–W–Cr alloy exhibits finer helium bubbles with a higher number density, leading to considerably reduced irradiation swelling. These results underscore that tailored compositional design is an effective route to mitigating irradiation-induced degradation in nickel-based alloys for advanced reactors.

### 2.3. Effect of Processing on Microstructure and Properties

As the core structural materials for advanced nuclear reactors, nickel-based alloys must endure the coupled extreme conditions of high temperature, stress, intense neutron irradiation, and corrosive media such as molten salts or high-temperature water. The long-term service stability of these alloys is governed not only by intrinsic attributes—including initial chemical composition, matrix phase constitution, microstructural configuration, grain size, and grain boundary character distribution—but also, to a large extent, by key processing parameters such as deformation temperature, strain rate, and imposed stress state [45]. These parameters directly regulate the flow behavior, dynamic/static recrystallization kinetics, and precipitation/dissolution of secondary phases during processing, thereby influencing the uniformity of the as-fabricated microstructure and the residual stress distribution, and ultimately dictating the evolution of irradiation-induced defects, the propensity for irradiation hardening/embrittlement, and the resistance to stress corrosion cracking in extreme service environments [46,47,48,49]. Importantly, even for nickel-based alloys of identical nominal composition, widely divergent key service properties can emerge from different processing routes and parameters, which firmly underscores the critical role of processing control in optimizing the service performance of reactor-grade nickel-based alloys.

#### 2.3.1. Hot Working

Hot working is a processing method conducted above the recrystallization temperature, during which the metal undergoes simultaneous plastic deformation and recrystallization. Figure 3 presents the investigation by Zhang et al. [50] into the microstructural evolution of GH4706 alloy across varied temperatures and strain rates, providing clear evidence that hot working profoundly influences the microstructural development of nickel-based alloys. Key parameters—including temperature, strain rate, and strain—directly dictate the resulting grain size, precipitate distribution, and ultimate mechanical properties. By carefully controlling these parameters, a fine, uniform grain structure with a well-tailored precipitate configuration can be achieved, thereby providing an optimal microstructural basis for downstream processing and further property enhancement [51,52]. To systematically evaluate hot workability, processing maps based on the dynamic material model (DMM) have been widely adopted. It has been shown that dynamic recrystallization (DRX) effectively refines the grain structure and reduces flow stress, thus improving the overall hot workability of nickel-based superalloys [53]. For instance, Liu et al. [54] investigated the hot deformation behavior of Incoloy 945 alloy via hot compression tests and identified markedly different DRX mechanisms depending on processing conditions: at large strains, both discontinuous DRX (DDRX) and continuous DRX (CDRX) are simultaneously activated; at low strain rates, the dynamic response of sub-grain-induced DRX (SDRX) intensifies with increasing deformation temperature; at high strain rates, DDRX gradually becomes dominant as temperature rises; and under coupled low-temperature/high-strain-rate conditions, geometric DRX (GDRX) is significantly activated at low strain levels. Furthermore, Sui et al. [55] reported that in the processing map of Inconel 718 at a strain of 10%, only one instability region (associated with adiabatic shear bands) and a small DRX domain in the safe region appear; however, once the strain exceeds 30%, three distinct instability regions emerge—one for adiabatic shear bands and the other two for intergranular cracking.

Consequently, by regulating dynamic recrystallization behavior and second-phase precipitation characteristics, hot working processes directly determine the grain size, grain boundary character distribution, and microstructural uniformity of nickel-based alloys, and these hot-working-induced microstructural features serve as the critical link between forming processes and in-service performance, ultimately governing the evolution of irradiation-induced defects, the propensity for irradiation hardening/embrittlement, and the stress corrosion cracking resistance of the alloys under the extreme service conditions of advanced nuclear reactors.

#### 2.3.2. Cold Working

Cold working refers to plastic deformation processes carried out below the recrystallization temperature, such as cold rolling, cold drawing, cold forging, stamping, and cold extrusion. Characterized by high deformation resistance, cold working shapes the metal while simultaneously enhancing its hardness and strength through work hardening, making it one of the core means of tailoring the microstructure and properties of nickel-based alloys. Extensive research has confirmed that the entire cold working process induces continuous and controllable microstructural evolution, which ultimately governs the final service performance [56,57,58,59,60,61]. For example, Wang et al. [62] reported that cold rolling caused grain fragmentation and elongated the microstructure along the rolling direction in C-276 alloy, whereas with rising annealing temperature the fibrous structure completely vanished and recrystallization occurred. Furthermore, our group has investigated the effect of cold rolling on the microstructure, mechanical properties, and corrosion behavior of Ni–17Mo–7Cr alloy, revealing that cold rolling significantly refines grains, increases dislocation density and lattice microstrain, and alters the dominant crystallographic texture, resulting in markedly increased hardness, yield strength, and tensile strength, but sharply reduced ductility [63]. These findings indicate that careful control of cold working and subsequent annealing parameters can tailor the microstructure, thereby providing a process-oriented foundation for enhancing the service performance of nickel-based alloys under the extreme environments of advanced nuclear reactors.

Cold working is a critical process for shaping and tailoring the properties of nickel-based alloys, during which the deformation parameters (including reduction, deformation mode, and workpiece dimensions) together with subsequent annealing jointly dictate the microstructural evolution pathway. Specifically, the cold deformation stage induces grain elongation and fragmentation, dislocation multiplication, progressive disappearance of annealing twins, and the formation of a fibrous structure, while simultaneously enhancing strength and hardness through work hardening. The annealing stage then eliminates the deformed microstructure through recrystallization, transforming the elongated grains into equiaxed ones, with the coupling between annealing temperature and prior cold deformation directly governing the recrystallized grain size, grain boundary character, and the resultant gradients in mechanical properties. By precisely matching the cold working parameters and annealing conditions, the microstructure can be controllably tailored to meet the performance demands of high-performance nickel-based alloys under extreme service scenarios such as those encountered in advanced nuclear reactors.

### 2.4. Challenges and Limitations

Overall, although nickel-based alloys show considerable promise for advanced nuclear reactors, their practical deployment still faces severe challenges in manufacturing, chemical compatibility, and irradiation stability, which directly impact long-term economic viability and operational safety. First, the fabrication of these alloys is inherently difficult: many nickel-based alloys, particularly highly alloyed grades, exhibit poor hot workability with high deformation resistance and a strong tendency to crack, rendering microstructural control demanding; this imposes stringent requirements on forging, welding, and other processes, and the frequent occurrence of defects such as cracks and inhomogeneity leads to low yields and sharply increased manufacturing costs [64]. Second, when exposed to high-temperature corrosive media such as molten fluoride salts, nickel-based alloys suffer from selective dissolution—Cr, as a principal alloying element, is preferentially dissolved due to the favorable thermodynamics of fluoride formation, which not only degrades corrosion resistance but can also induce near-surface voiding that accelerates structural integrity deterioration [65]. Third, irradiation-induced microstructural evolution constitutes the core threat to safe service: on the one hand, high-energy neutron irradiation promotes point-defect accumulation that evolves into dislocation loops and voids, causing severe swelling; on the other hand, the transmutation of nickel generates substantial He atoms that preferentially nucleate and grow as bubbles at grain boundaries, leading to helium embrittlement and a significant loss of ductility and toughness [66,67,68,69]. Furthermore, irradiation also induces solute segregation, promotes precipitation of brittle phases, and causes irradiation hardening and accelerated creep, which collectively result in severe property degradation after prolonged exposure [70]. Therefore, overcoming manufacturing-induced defects, enhancing high-temperature corrosion stability, and suppressing the formation and evolution of irradiation-induced defects are the principal technical barriers that must be addressed for the practical application of nickel-based alloys in advanced reactors.

## 3. Irradiation Damage Behavior of Nickel-Based Alloys

Under the extreme irradiation environment of advanced nuclear reactors, structural materials are simultaneously subjected to high temperature and high-dose neutron irradiation; when high-energy neutrons collide with lattice atoms, they transfer part of their energy to the struck atoms, creating primary knock-on atoms (PKAs) that subsequently trigger a cascade of further atomic collisions—the fundamental mechanism of irradiation displacement damage. This cascade process not only generates a high concentration of Frenkel pairs but also initiates a complex sequence of microstructural evolution that ultimately leads to macroscopic property degradation [71,72]. Typically, a collision cascade evolves through four distinct stages: (I) the initial collisional phase, (II) the thermal spike phase, (III) the defect recombination/annealing phase, and (IV) the steady-state phase, with temperature playing a decisive role in modulating defect survival and clustering during the latter stages. Figure 4 illustrates the time-resolved evolution of point defects in metallic Ni during such a cascade process [73]. Building upon this conceptual foundation, the following sections will systematically elucidate the microscopic formation mechanisms of irradiation damage in nickel-based alloys and reveal how these mechanisms govern the degradation of their macroscopic properties.

### 3.1. Microstructural Defect Evolution

Collision cascades generate a high concentration of Frenkel pairs (vacancies and interstitials), which subsequently diffuse and aggregate to form more complex defect clusters characteristic of nickel-based alloys, such as stacking-fault tetrahedra (SFTs), dislocation loops, and voids [74,75,76,77,78]. When transmutation helium is present, vacancies preferentially trap He atoms, leading to the nucleation and growth of helium–vacancy complexes that eventually evolve into helium bubbles [79].

#### 3.1.1. Dislocation Loops and Voids

After collision cascades, most point defects recombine through interstitial–vacancy annihilation, while surviving vacancies migrate and aggregate into three-dimensional voids that cause irreversible irradiation swelling, and un-recombined interstitials cluster to form dislocation loops. The formation of voids, which underlies macroscopic swelling, has been rationalized through several complementary mechanisms: dislocation bias, whereby dislocations preferentially absorb interstitials over vacancies, leaving an excess of vacancies to aggregate into voids; cascade localization-induced bias, in which the initial spatial separation of interstitials and vacancies within a collision cascade favors vacancy accumulation; and production bias, where one-dimensionally migrating interstitial clusters produced directly in cascades escape their birth region, resulting in a net vacancy supersaturation [80,81,82]. These irradiation-induced loops typically nucleate directly from cascade debris as small clusters and subsequently grow by absorbing point defects, eventually interacting to form complex dislocation networks and reaching a stable configuration dominated by specific Burgers-vector types, with only minor morphological adjustments thereafter [83]. For example, He et al. [84] simulated the nucleation and growth of dislocation loops and voids in Hastelloy C276 under Ar^+^ irradiation using the multiscale code RADIEFF, and found that loop size increases with dose and is further promoted by temperature; owing to the much higher diffusivity of interstitials compared to vacancies, loops grow faster than voids, and void growth becomes increasingly sluggish as their size increases, as shown in Figure 5. Given the compositional complexity of nickel-based alloys, isolating the role of a specific solute is challenging. Through combined experiments and simulations, Ma et al. [85] first revealed the influence of solute type, concentration, and temperature on dislocation loop properties in FCC Ni: large Ti atoms tend to segregate at strained lattice sites around irradiation defects, reducing loop mobility and decreasing the stacking-fault energy of Ni. With increasing Ti content, the fraction of Frank loops and the size and density of SFTs rise; above a critical Ti concentration, interstitial-type loops dominate, while below it vacancy-type loops prevail, and the critical concentration decreases with increasing temperature. Furthermore, Shan et al. [86] reported that higher dose and temperature accelerate the unfaulting of Frank loops, which can mitigate irradiation hardening, whereas chemically complex systems may stabilize Frank loops by retarding defect diffusion. Hence, the introduction of specific alloying elements can effectively retard the growth kinetics of dislocation loops, thereby enhancing irradiation resistance.

#### 3.1.2. Phase Transformations and Precipitation

At the chemical level, irradiation can also profoundly affect phase transformations and precipitation behavior, as schematically summarized in Figure 6. The high concentration of point defects created by energetic particle bombardment greatly accelerates solute diffusion, driving non-equilibrium phase transitions [87,88]; in nickel-based alloys, this promotes the formation of precipitates enriched in specific elements, with distinct morphologies and spatial distributions. These precipitates typically consume critical solid-solution strengthening elements from the matrix, leading to a weakening of the original solid-solution hardening. Under the synergistic effects of high temperature and irradiation, enhanced diffusion further redistributes solute atoms, providing the kinetic conditions for precipitate nucleation and growth, so that both the number density and the average size of precipitates increase with dose. Moreover, precipitate–matrix interfaces act as effective sinks for irradiation-induced vacancies and transmutation helium atoms, causing helium bubbles and voids to preferentially nucleate and grow at these interfaces, which in turn accelerates irradiation swelling [89]. An illustrative mechanical response is provided by nanoindentation of GH3617 alloy: in unirradiated and low-fluence He-ion irradiated (1 × 10^16^ cm^−2^) samples, the nanohardness in the near-surface region (<250 nm) decreased gradually with increasing depth, whereas at higher fluences (5 × 10^16^ and 1 × 10^17^ cm^−2^) the hardness exhibited an anomalous abrupt drop followed by a recovery [90]. This phenomenon can be consistently interpreted as follows: high-dose He irradiation creates a soft, bubble-rich layer very close to the surface due to pronounced void and helium-bubble formation, while the underlying zone is hardened by irradiation-induced defects (dislocation loops and precipitates). Hence, as the indenter penetrates through the softened surface layer into the hardened subsurface region, the measured hardness first plummets and then rises, reflecting the complex interplay between irradiation-induced precipitation, void formation, and mechanical properties.

#### 3.1.3. Helium Bubbles

Under intense neutron irradiation, Ni atoms undergo (n,α) transmutation reactions, producing substantial helium that accumulates at grain boundaries and phase interfaces to form bubbles, resulting in helium embrittlement and swelling [91]. As demonstrated by Zhu et al. [92], in Inconel 617 irradiated with 180 keV He ions at RT and subsequently annealed at 700 °C, helium bubbles underwent long-range migration, with those in the He concentration and damage peak region transforming from spherical to faceted morphologies; this coarsening was attributed to migration and coalescence, while the presence of solute elements retarded such behavior, thereby enhancing resistance to helium embrittlement. Typically, with increasing dose, bubble size continuously grows; at intermediate doses the number density rises markedly, but at high doses it drops sharply due to coalescence, as mobile bubbles aggregate into larger ones. Figure 7 displays the helium bubble distributions within the helium-concentration peak region for three variants of Inconel 718 alloy exposed to different irradiation doses, revealing a progressive enlargement of bubbles with increasing dose and a pronounced dose-dependent evolution [93]. Notably, although the transition from low to intermediate doses induces pronounced swelling, the further evolution toward high doses does not suppress it; rather, the total swelling continues to increase because the volume fraction of helium bubbles keeps growing, even as number density declines. Dislocations act as preferential trapping sites for He atoms, promoting the nucleation of large bubbles along dislocation lines, often at low-energy positions where bubbles align. For example, through dual-beam H_2_^+^/He^+^ irradiation of GH3535 alloy, Zhu et al. [94] showed that irradiation-induced interstitials and He atoms preferentially nucleate at dislocation loops, accelerating loop growth while forming helium bubble–dislocation loop complexes, and that the bubble number density initially rises rapidly toward saturation with gradual coarsening before coalescence among adjacent bubbles leads to a decline in number density and a concomitant significant increase in mean bubble size.

### 3.2. Macroscopic Property Degradation

The extreme irradiation environment in advanced nuclear reactors induces the evolution of defect clusters in nickel-based alloys, which ultimately manifests as several typical degradation phenomena—including irradiation hardening, embrittlement, swelling, and creep—thereby severely threatening the service life and structural integrity of the materials.

#### 3.2.1. Irradiation Hardening

Irradiation hardening, one of the most prominent macroscopic property changes under irradiation, stems from the obstruction of dislocation motion by irradiation-induced point-defect clusters, dislocation loops, and helium bubbles, as illustrated in Figure 8. The dominant hardening mechanism evolves dynamically with dose and temperature: at low He-ion fluences, Lv et al. [95] found that vacancy-type defects in Inconel 617 produce only small microstrains, leading to a negligible hardness increase. By contrast, Yang et al. [96] reported that in GH3535 alloy the hardness saturates at approximately 0.5 dpa and remains nearly constant with further dose increase (Table 4), with hardening governed by the interaction between gliding dislocations and irradiation-induced dislocation loops. Interestingly, at 700 °C and a high dose of 8.0 dpa, the hardness is significantly greater than that at RT, and the dominant mechanism shifts to strengthening by a high density of homogeneously dispersed irradiation-induced precipitates, highlighting that irradiation hardening is also strongly temperature-dependent.

Beyond the hardening contributions from dislocation-type defects, precipitation and helium bubble evolution also play significant roles. For instance, in GH3535 alloy, irradiation-induced (Mo,Cr)-rich precipitates constitute a key hardening factor; although these precipitates tend to nucleate preferentially at irradiation-produced dislocation loops, the accompanying solute enrichment substantially increases the obstacle strength, thereby further enhancing the hardening effect [97]. On the other hand, helium bubbles in Hastelloy N irradiated at 650 °C can also act as strong obstacles to dislocation glide, though their strength is somewhat lower than that of displacement damage defects such as dislocation loops and point-defect clusters formed under saturated heavy-ion irradiation, while voids at this temperature contribute negligibly to hardening [98]. Collectively, these findings demonstrate that the dominant hardening mechanisms vary significantly with alloy system and irradiation conditions.

#### 3.2.2. Irradiation Embrittlement

Irradiation embrittlement, a typical degradation phenomenon that usually occurs concomitantly with irradiation hardening, manifests as a significant loss of ductility, an upward shift in the ductile-to-brittle transition temperature, increased yield strength, and reduced elongation, ultimately promoting intergranular fracture that severely threatens the long-term integrity of reactor structural components [99]. Generally speaking, as irradiation damage accumulates, the embrittlement effect becomes increasingly severe. Roy et al. [100] demonstrated that, for both PM-HIP and forged Ni-based Alloy 625, higher damage levels cause the fracture surfaces to exhibit a more brittle fracture character with a marked reduction in dimples, as illustrated in Figure 9. At the microstructural level, the nucleation and growth of helium bubbles at grain boundaries constitute a critical embrittlement mechanism: He atoms produced by transmutation reactions migrate and aggregate into helium–vacancy clusters that evolve into stable bubbles. Jin et al. [90] demonstrated that in GH3617 alloy subjected to high-dose He-ion irradiation, helium bubbles preferentially precipitate and coarsen along grain boundaries, drastically reducing grain-boundary cohesion and causing intergranular fracture. Similarly, Griffiths [101] reported that in the fatigue testing of Inconel X-750, unirradiated material exhibited ductile fracture, whereas irradiated material showed brittle intergranular fracture due to grain-boundary helium bubble accumulation. In addition to helium effects, irradiation-induced dislocation loops and SFTs impede dislocation motion, raising the yield strength while decreasing elongation. Concurrently, the high concentration of irradiation-produced point defects enhances solute diffusion, leading to grain-boundary segregation and, in some cases, the formation of precipitates enriched in specific alloying elements, which further elevate the ductile-to-brittle transition temperature. The synergistic interplay among these mechanisms constitutes the overall irradiation embrittlement process in nickel-based alloys.

#### 3.2.3. Irradiation Swelling and Creep

Irradiation swelling is a macroscopic volume expansion (i.e., density reduction) exhibited by reactor structural materials under neutron irradiation, which not only acts synergistically with irradiation embrittlement, hardening, and creep to degrade mechanical integrity but also originates from the dynamic evolution and accumulation of irradiation-induced defects [102,103]. For instance, Jin et al. [90] showed that in GH3617 alloy, with increasing irradiation temperature, the number density of irradiation-induced voids decreases while their mean size increases significantly. This evolution is further promoted by radiation-enhanced diffusion, which accelerates solute migration and redistribution, thereby driving the nucleation and continuous growth of precipitates. In nickel-based alloys, the presence of complex precipitates such as M_23_C_6_ and γ′ provides a high density of incoherent or semi-coherent interfaces that act as synergistic traps for both vacancies and transmutation helium atoms. Owing to the strong binding energy between helium and vacancies, He atoms that migrate to these interfaces stabilize and accelerate the nucleation and growth of voids, causing the overall swelling to intensify continuously with increasing irradiation dose.

Irradiation creep refers to the enhanced creep deformation that occurs under irradiation, driven by the stress-induced asymmetric absorption of irradiation-produced point defects by dislocations, which facilitates dislocation climb and enables significant creep strain at stresses below the thermal creep threshold. The mechanisms are generally classified into two categories: the first attributes creep strain directly to the climb of dislocations resulting from stress-induced preferential nucleation (SIPN) or stress-induced preferential absorption (SIPA) of point defects; the second considers that creep strain arises from dislocation glide, with the necessary climb step being driven by point-defect absorption, as described by the I-creep model proposed by Gittus [104]. In practical irradiation environments, these two types of mechanisms often operate concurrently and are coupled, collectively dictating the macroscopic creep behavior under varying conditions of temperature, stress, and irradiation dose. Recently, Ning et al. [105] developed a new steady-state irradiation creep constitutive model based on non-equilibrium statistical mechanics and validated it against in-reactor creep data for Hastelloy N, as shown in Figure 10. Using this model to predict the irradiation creep rupture time of Hastelloy N in the temperature range of 600–800 °C, stress range of 100–350 MPa, and neutron fluence range of 1.3 × 10^19^–1.5 × 10^21^ n/cm^2^, they revealed for the first time the nonlinear behavior of the creep life reduction factor. A thorough understanding of these microscopic mechanisms and their interplay is therefore essential for the accurate prediction of the long-term service performance of reactor structural materials.

## 4. Methods for Studying Irradiation Damage

Driven by the demanding performance requirements of advanced nuclear energy systems, researchers have developed and integrated a wide range of experimental and computational methods over recent decades to systematically investigate the microstructural evolution and macroscopic property degradation of nickel-based alloys under irradiation. These approaches can be broadly divided into experimental characterization and computational modeling, which complement and validate each other to provide a multi-scale understanding of the irradiation damage mechanisms in nickel-based alloys.

### 4.1. Experimental Methods

Experimental research aims to simulate the actual service irradiation environment of materials, and by employing advanced characterization techniques, it enables the direct observation of microstructural evolution and the measurement of macroscopic property changes; according to their specific purposes, these experimental methods can be categorized into three main groups: irradiation experiments, microstructural characterization, and macroscopic property testing.

#### 4.1.1. Irradiation Techniques

Ion irradiation, characterized by high energy density, precisely controllable experimental conditions, and the absence of solid-solubility constraints, has become the most widely used surrogate for neutron irradiation in fundamental studies. It enables the introduction of well-controlled displacement damage and, when desired, the co-injection of transmutation-relevant species such as He and H. Compared with reactor neutron irradiation, ion irradiation offers distinct advantages—short timescales, low cost, and no induced radioactivity—making it highly compatible with other characterization techniques for mechanistic investigations. For example, Zhu et al. [70] employed 1.2 MeV H^+^ ions at 650 °C to simulate the service conditions of GH3535 alloy in molten salt reactors and successfully elucidated the evolution mechanism of Ni_3_Si precipitates and the anisotropic behavior of Frank dislocation loops. Similarly, Jin et al. [90] irradiated GH3617 alloy with 0.5 MeV He^2+^ at RT, 500 °C, and 800 °C to investigate the synergistic evolution of M_23_C_6_ precipitates and voids, and their impact on void swelling and mechanical properties. Despite inherent differences such as high dose rate and limited ion penetration depth, ion irradiation remains an indispensable tool for probing the underlying mechanisms of irradiation damage.

#### 4.1.2. Microstructural Characterization

After irradiation, samples typically require microstructural characterization using techniques such as transmission electron microscopy (TEM), scanning electron microscopy (SEM), and atom probe tomography (APT), often combined with energy-dispersive X-ray spectroscopy (EDS). TEM enables direct observation of crystal defects—including dislocations, stacking faults, twins, precipitates, voids, and dislocation loops—and when coupled with EDS, it provides elemental composition and distribution information, making it the core tool for irradiation damage research; Figure 11 presents TEM images of Hastelloy N at different irradiation doses [106]. SEM, particularly field-emission SEM, can achieve a spatial resolution better than 1.0 nm under optimized conditions and is mainly used for surface morphology imaging, while grain orientation and compositional mapping are realized by equipping it with electron backscatter diffraction (EBSD) and EDS detectors, respectively [107]. Moreover, the rapid development of focused ion beam (FIB) micromachining for site-specific sample preparation has greatly enhanced the accuracy and relevance of both TEM and APT analyses. For example, Zhu et al. [70] combined APT and TEM to investigate the nucleation and evolution of Ni–Si clusters and Ni_3_Si precipitates in GH3535 alloy, providing direct evidence for the formation of Cr/Mo-rich M_2_C carbides and the absence of Si segregation at Frank dislocation loops.

#### 4.1.3. Macroscopic Property Testing

For ion-irradiated materials, irradiation-induced microstructural changes ultimately manifest in mechanical properties, and nanoindentation has become the predominant technique for evaluating these properties due to its ability to probe shallow damage layers. By applying a minute load with a diamond indenter and continuously recording the penetration depth, a load–displacement curve is established from which hardness and elastic modulus are derived [108]; the technique can also assess fracture toughness, creep behavior, and yield stress at small scales, with relatively minimal sample preparation. Owing to the limited thickness of the irradiated layer, nanoindentation predominantly samples the damaged region, and when operated in continuous stiffness measurement (CSM) mode, it yields depth-resolved mechanical data, allowing the substrate effect to be decoupled through suitable models (see Figure 12). For example, Song et al. [109] employed nanoindentation to reveal that the combined strengthening of MC–M_23_C_6_ carbides in Haynes 282 alloy outperforms that of single carbide phases—a capability equally valuable for investigating irradiation-induced precipitates. In a direct irradiation context, Zhu et al. [110] applied CSM nanoindentation to characterize the competing hardening from irradiation defects and softening from γ″ disordering in ion-irradiated Inconel 718.

### 4.2. Simulation and Modeling

Computational modeling employs theoretical constructs to perform numerical simulations, enabling atomic-scale elucidation of the microscopic mechanisms of irradiation damage and providing fundamental theoretical interpretations for experimental observations. By capturing transient processes such as collision cascades that are difficult to directly observe experimentally, and by allowing independent control over extreme conditions, it has become an indispensable and powerful tool in irradiation damage research. Depending on the spatial and temporal scales of interest, a hierarchy of methods has been developed, ranging from first-principles calculations and molecular dynamics to kinetic Monte Carlo and rate theory.

#### 4.2.1. First-Principles Calculations

First-principles calculations, grounded in quantum mechanics and free of empirical parameters, predict the electronic structure and associated properties of materials by solving the Kohn-Sham equations to treat electron–electron and electron–ion interactions from first principles. With density functional theory (DFT) as the central framework, this approach can yield—with high accuracy—fundamental physical quantities such as ground-state energies, electronic structures, defect formation energies, and migration barriers, thus establishing a rigorous theoretical foundation for unraveling the microscopic mechanisms of irradiation damage in nickel-based alloys.

First-principles calculations have been extensively employed to investigate irradiation-induced defect formation and evolution in nickel-based alloys by determining key energetic parameters such as defect formation energies, migration barriers, and binding energies, thereby uncovering the intrinsic mechanisms governing irradiation resistance. For example, Liu et al. [111] demonstrated through DFT calculations that the presence of Nb in GH3535 alloy suppresses the binding of He atoms with vacancies and reduces their diffusivities, consequently impeding the nucleation and growth of helium bubbles; additionally, Nb promotes the formation of irradiation-induced superlattice precipitates, which raises the alloy’s hardness. Although the precipitate strengthening contributes to hardening, the inhibition of helium bubble formation markedly enhances the overall resistance to helium embrittlement, highlighting the beneficial role of Nb. Such predictive capability of first-principles methods in assessing the influence of alloying elements on defect behavior provides direct theoretical guidance for the composition design of irradiation-tolerant nickel-based alloys [112,113].

#### 4.2.2. Molecular Dynamics Simulations

Molecular dynamics (MD) simulations, based on the principles of classical Newtonian mechanics, numerically solve the equations of motion for atoms interacting through empirical interatomic potentials to obtain their trajectories (i.e., the time evolution of atomic positions and velocities), thereby capturing the key microscopic processes of irradiation damage at the atomic scale—such as collision cascades, point-defect generation and diffusion, and the evolution of dislocation loops; the reliability of such simulations depends critically on the accuracy of the chosen potential. These calculations are typically performed using large-scale parallel software like the LAMMPS [114], and the resulting atomic configurations are analyzed and visualized with tools such as OVITO to characterize defect structures [115].

In the irradiation damage research of nickel-based alloys, MD simulations are primarily used to unravel the atomic-scale evolution mechanisms of defects, thereby providing microscopic insights into their correlation with macroscopic properties. For example, using MD simulations with a modified embedded-atom method (MEAM) potential, Esfandiarpour et al. [116] examined defect formation and evolution in Inconel 617 subjected to single and successive overlapping cascades up to 0.2 dpa (see Figure 13); their work revealed that, although the defect concentration is similar to that reported for equimolar NiCrCo, the alloy generates fewer dislocations and smaller defect clusters, indicating enhanced radiation tolerance at this damage level. Zhu et al. [70] employed MD to confirm the enrichment distribution mechanism of Ni and Si at dislocation loop edges revealed by APT, and to reveal that compressive stress from ion penetration leads to a distinct increase in the formation energy of four Frank loop variants. In another study on Cu–Ni alloys, Wei et al. [117] employed MD to explore the effects of temperature, PKA energy, crystal orientation, and Ni content on displacement cascade evolution, systematically analyzing the influence of irradiation on microstructure and mechanical properties, and proposed that a higher Ni content plays a crucial role in enhancing irradiation resistance and maintaining mechanical stability. The predictive power of such simulations is heavily dependent on the accuracy of the interatomic potentials, and the recent rapid development of embedded atom method (EAM) and machine-learning potentials tailored for nickel-based alloys has substantially improved the reliability of MD results [118,119,120,121]. It should be emphasized that these machine-learned interatomic potentials, trained on extensive first-principles databases, approach DFT accuracy for defect energetics at orders-of-magnitude lower computational cost, enabling statistically robust sampling of rare defect reaction events—such as helium bubble nucleation and loop–dislocation interactions—that govern the long-term evolution of irradiation damage.

#### 4.2.3. Kinetic Monte Carlo Simulations

Kinetic Monte Carlo (KMC) is a stochastic simulation method based on the master equation of Markov processes, in which the temporal evolution of a system is represented as a sequence of random transitions between discrete states, with each transition rate determined by the corresponding rate constant [122,123]. Unlike MD, which relies on deterministic Newtonian dynamics, KMC advances the system through stochastic sampling, thereby extending the accessible timescale from nanoseconds to seconds or even longer while retaining atomic-level resolution. The method is generally divided into two major categories: atomistic KMC (AKMC), in which individual atomic jumps on a lattice are treated as elementary events, and object KMC (OKMC), where the elementary objects are abstract entities such as vacancies, interstitials, or defect clusters, enabling significantly higher computational efficiency [124,125].

KMC, especially OKMC, has become an indispensable tool for studying the long-term evolution of irradiation damage in nickel-based alloys, primarily because it spans the timescales between short-term MD simulations and experimental observations. In practice, KMC is often coupled with MD: collision cascades are first simulated by MD to generate an initial defect distribution, which is then used as input for KMC to track the migration, clustering, and recombination of vacancies and interstitials over much longer timescales. For instance, by incorporating initial cascade defect configurations and migration barriers from MD, Xiao et al. [126] demonstrated that OKMC bridges timescales from nanoseconds to seconds, enabling systematic simulation of the long-term annealing evolution of point defects and their clusters in nickel. This approach effectively reveals the recombination, migration, and residual defect mechanisms under different cascade temperatures and PKA energies, overcoming the inherent temporal limitation of pure MD simulations. In another study, Ferasat et al. [127] developed a parameterized OKMC framework to simulate helium bubble aggregation and growth in pure nickel during and after helium implantation, showing that the average bubble size increases with temperature but decreases with implantation dose rate, and highlighting the crucial role of defect–interface interactions in controlling bubble size and stability.

#### 4.2.4. Rate Theory

Rate theory is a widely used computational approach in irradiation damage research, which treats defect species as spatially homogeneous concentrations and describes their production, recombination, and migration through a set of coupled ordinary differential equations. Owing to its high computational efficiency, rate theory is well suited to predicting the long-term evolution of macroscopic properties such as swelling and creep, although it inherently neglects spatial correlations and local microstructural fluctuations within a mean-field approximation. For example, Yoo et al. [128] incorporated the partitioning of mobile helium and the trapping of interstitial helium by vacancies into a rate theory model to predict swelling induced by void and bubble growth in nickel-based materials. Their results demonstrated that, compared with simultaneous helium injection during heavy ion bombardment, helium pre-implantation is insufficient to fully capture the actual service conditions of fusion reactor environments, and that dislocation density and grain size significantly affect the predicted swelling behavior.

## 5. Strategies for Irradiation Resistance

In response to the severe challenges imposed by the extreme environments in advanced nuclear reactors, a variety of strategies have been developed in recent years to enhance the irradiation resistance of nickel-based alloys—centering on alloying element selection and doping, microstructural design, and composite structural material design—thereby effectively suppressing the generation and evolution of irradiation-induced defects by tailoring chemical composition and microstructure, thereby substantially improving both irradiation tolerance and long-term service life.

### 5.1. Alloying Elements

The precise selection and doping of alloying elements is a fundamental strategy for optimizing the irradiation resistance of nickel-based alloys. By introducing specific elements to tailor the microstructure, researchers can effectively suppress the nucleation and growth of irradiation-induced defects—such as dislocation loops and helium bubbles—thereby mitigating irradiation hardening, swelling, and embrittlement [129,130]. Numerous studies have shown that different alloying elements influence defect evolution through distinct microscopic mechanisms. For instance, Wei et al. [44] employed density functional theory calculations to reveal that Mo and W inhibit helium bubble coarsening by enhancing lattice stability and increasing the He migration barrier, whereas Cr attracts He atoms via orbital hybridization and electron cloud overlap, acting as additional trapping centers that promote bubble nucleation; this leads to a finer, more dispersed bubble distribution, and experiments by Li et al. [131] confirmed that introducing a certain amount of Cr into Ni–16Mo alloy effectively retards bubble growth. Cao et al. [132] further demonstrated that Mo addition causes irradiation hardening to saturate earlier and significantly reduces defect size and density compared to pure Ni (see Figure 14). Moreover, recent advances in multi-principal element alloy strategies (e.g., high-entropy alloy concepts) have shown that severe lattice distortion and complex local chemical environments can profoundly alter defect diffusion and recombination behaviors [133,134,135]; systematic comparisons have revealed that the chemical disorder in high-entropy alloys effectively suppresses the formation and aggregation of irradiation defects, resulting in much smaller dislocation loops, exceptional phase stability, strong resistance to irradiation-induced segregation, and extremely low swelling rates [136,137].

Future alloy design is anticipated to move beyond minor compositional modifications of conventional nickel-based alloys and progressively embrace multi-principal element high-entropy alloys; accordingly, research will focus on synergistically exploiting the inherent compositional complexity of high-entropy alloys—such as sluggish diffusion and severe lattice distortion—together with tailored interface engineering to develop next-generation irradiation-resistant nickel-based alloys with self-healing characteristics, a paradigm that not only provides new perspectives on irradiation damage mechanisms but also establishes a solid scientific foundation for the development of structural materials capable of meeting the extreme service demands of Gen-IV nuclear reactors.

### 5.2. Microstructural Design

In recent years, microstructural design has proven to be an effective strategy for enhancing the irradiation resistance of nickel-based alloys. Among the various microstructural features, grain boundaries and dislocations act as two typical defect sinks that play a pivotal role in the evolution of irradiation-induced defects. This section therefore focuses on elucidating the effects and underlying mechanisms of high-density dislocations and grain boundary engineering on the irradiation resistance of nickel-based alloys.

High-density dislocations can serve as efficient sinks for irradiation-induced point defects, promoting their recombination and thereby suppressing damage accumulation. In terms of dislocation engineering, Roy et al. [100] attributed the greater irradiation hardening observed in forged Alloy 625 (or Alloy 690) relative to its PM-HIP counterpart to the higher density of pre-existing dislocations, which serve as preferential sinks for interstitials and thereby promote vacancy supersaturation and void formation, resulting in more pronounced hardening at both damage levels, as depicted in Figure 15. Bazarbayev et al. [138] introduced a high density of dislocations into the near-surface region of Inconel 718 via LSP and ultrasonic nanocrystal surface modification (UNSM), effectively mitigating the increase in defect density and the disordering of second phases under irradiation. Furthermore, Ni et al. [139] demonstrated that, compared to untreated Inconel 718, LSP-treated alloy exhibited a 40.8% reduction in the dissolution rate of second phases, significant inhibition of dislocation loop growth, and a 26.7% decrease in irradiation hardening. These findings highlight the critical role of pre-existing high-density dislocations in enhancing the irradiation tolerance of nickel-based alloys.

With respect to grain boundary engineering, He et al. [91] reported that GH3535 alloy fabricated by powder metallurgy and hot isostatic pressing possesses significantly refined grains (i.e., higher grain boundary density), which markedly enhances helium trapping at grain boundaries, thereby inhibiting helium bubble nucleation and growth and ultimately delaying irradiation swelling. Beyond increasing grain boundary area, optimizing the grain boundary character distribution—specifically, raising the fraction of low-Σ coincidence site lattice (CSL) boundaries—has also been proven effective in boosting irradiation resistance; for example, Tan et al. [140] applied thermomechanical processing to Incoloy 800H and Inconel 617 alloys to achieve a high proportion of special boundaries, and after irradiation these alloys exhibited not only superior high-temperature yield strength and ultimate tensile strength but also notably improved corrosion resistance compared to their untreated counterparts.

Overall, future strategies for enhancing the irradiation resistance of nickel-based alloys should focus on microstructural tailoring—particularly the synergistic optimization of grain boundary character and the introduction of high-density dislocations—to manipulate the behavior of point defects, dislocation loops, and helium diffusion/trapping/bubble evolution, thereby enabling the development of novel nickel-based alloys with exceptional long-term stability and durability for advanced nuclear reactors.

### 5.3. Composite Structural Materials

In advanced nuclear reactor environments, composite structural materials—fabricated by incorporating a high density of stable nano-oxides, carbides, or engineering layered/graded architectures within the nickel-based matrix—introduce abundant phase boundaries that act as effective sinks for irradiation-induced point defects and helium atoms while pinning dislocations, thereby synergistically suppressing defect accumulation, swelling, and embrittlement, and opening a promising design pathway for next-generation irradiation-resistant nickel-based alloys.

Nanoscale oxide dispersion-strengthened (ODS) nickel-based alloys, which incorporate a high density of uniformly dispersed nano-oxides (e.g., Y_2_O_3_, Al_2_O_3_) into the matrix, represent a promising route to enhance irradiation resistance by creating abundant matrix/oxide heterogeneous interfaces. In Y_2_O_3_-reinforced Ni–Mo–Cr alloys, these interfaces preferentially trap interstitials, leaving excess vacancies that bind with helium to form stable He–vacancy complexes, thereby providing a high density of nucleation sites for helium bubbles; this results in a refined dispersion of fine bubbles with significantly smaller diameters than those in conventional Hastelloy N alloy [141], effectively suppressing bubble coarsening and associated embrittlement. Moreover, Li et al. [142] demonstrated that under coupled He^+^ irradiation and FLiNaK molten salt corrosion, the NiMoCr/Y_2_O_3_ interfaces not only trap Cr atoms that would otherwise dissolve into the salt, forming a Y_2_O_3_–Cr_2_O_3_ composite layer that retards Cr outward diffusion, but also suppress helium bubble growth by trapping He atoms (see Figure 16), highlighting the multifaceted benefits of interface engineering in ODS alloys.

Meanwhile, heterogeneous interface engineering—embodied in laminated or nano-dispersed composite architectures—has emerged as one of the most promising strategies for advanced reactor materials. Such structures provide high-density interfaces that act as efficient traps and recombination channels for irradiation-induced point defects, helium bubbles, and dislocation loops. For instance, our atomistic simulations of Ni/graphene nanocomposites revealed that, although graphene itself suffers progressive irradiation damage with increasing dose, the Ni/graphene interface exhibits a continuously enhanced defect-trapping capability; this enhancement originates from the damaged regions in graphene offering abundant recombination and annihilation sites for irradiation defects, thereby strengthening both the energetic and kinetic driving forces for defect capture—uncovering a novel interface-enabled self-healing mechanism for irradiation damage [143]. Complementary simulations further showed that Ni/graphene interfaces rapidly trap He atoms, which can migrate freely along the interface without chemical binding, effectively suppressing helium bubble growth and providing theoretical guidance for the design of helium-embrittlement-resistant composites [144]. Beyond two-dimensional layered systems, metal/ceramic nanocomposites also show unique advantages in thermal stability and helium resistance: Wei et al. [145] conducted in-situ TEM observations of a Ni/SiOC nanocomposite under He ion irradiation at 300 °C and found that the amorphous SiOC ceramic phase exhibits excellent structural stability, effectively inhibiting grain coarsening of the nickel matrix, while no noticeable helium bubble accumulation or large voids were detected within the amorphous phase or at the phase boundaries (Figure 17). These results demonstrate that the incorporation of an amorphous SiOC ceramic phase can effectively control the spatial distribution of He atoms, significantly enhancing the irradiation resistance of nickel-based alloys.

Thus, the core design strategy for composite structural materials lies in introducing abundant defect recombination centers and helium trapping sites, whereby heterogeneous interfaces effectively regulate the diffusion, recombination, and clustering of point defects and He atoms, thereby substantially mitigating irradiation embrittlement, hardening, and swelling. Future research should focus on developing novel, highly stable composite architectures with tailored high-density interfaces to meet the long service-life requirements of core components in Gen-IV nuclear reactors.

## 6. Summary and Outlook

In summary, structural materials for advanced reactors must maintain long-term stability under the synergistic effects of high temperature, high-dose irradiation, corrosive media, stress, and transmutation gases. Nickel-based alloys, with their FCC matrix, highly alloyed compositions, and exceptional high-temperature strength, oxidation resistance, and corrosion resistance, have become prime candidate structural materials for Gen-IV nuclear energy systems such as MSRs and VHTRs. Focusing on representative alloy systems including Hastelloy N/GH3535, Inconel 718, and Inconel 617, this review has systematically examined their service environments, key performance metrics, fabrication processes, irradiation damage behaviors, research methodologies, and anti-irradiation design strategies. Existing studies demonstrate that irradiation damage in nickel-based alloys originates from point defect generation, migration, recombination, and clustering induced by collision cascades, which further evolve into complex microstructural changes such as dislocation loops, voids, helium bubbles, irradiation-induced precipitation/phase transformations, and solute redistribution, ultimately manifesting as macroscopic property degradation—hardening, embrittlement, swelling, and reduced creep life—that critically limits the structural integrity and lifetime assessment of core components. Future efforts should focus on deepening the understanding of damage mechanisms under coupled extreme conditions, advancing multi-scale characterization and simulation techniques, and achieving breakthroughs in irradiation-resistant nickel-based alloys through compositional optimization, microstructural engineering, and composite material design, so as to meet the demanding long-service requirements of next-generation advanced nuclear reactors.

To address these challenges, the performance optimization of nickel-based alloys must shift from single compositional tuning toward the synergistic design of composition, microstructure, interfacial architecture, and processing. At the compositional level, alloying elements such as Mo, W, Cr, Nb, and Y can modulate defect formation energies, helium migration barriers, precipitate stability, and grain-boundary behavior to influence the evolution of dislocation loops and helium bubbles; meanwhile, high-entropy or multi-principal element alloys, leveraging their inherent chemical disorder and severe lattice distortion, provide new avenues to retard defect diffusion and suppress irradiation-induced segregation. Microstructurally, thermomechanical processing, cold working, and annealing enable precise control over dynamic recrystallization, grain size, dislocation density, and grain-boundary character distribution, whereas advanced strategies—including LSP, UNSM, grain boundary engineering, oxide dispersion strengthening, and heterogeneous interface architectures—can further construct high-density defect sinks and helium trapping sites, thereby delaying irradiation-defect accumulation, bubble coarsening, and interfacial degradation. Despite these advances, key engineering bottlenecks remain for nickel-based alloys, notably poor workability (cracking, welding difficulties, and microstructural inhomogeneity), selective Cr dissolution in high-temperature molten salts, an insufficient mechanistic understanding of irradiation–corrosion–stress coupling, and a critical lack of long-term neutron irradiation data to support reliable component qualification.

Therefore, future research should more closely replicate actual service environments. Experimentally, emphasis should be placed on establishing the quantitative equivalence and limitations between neutron and ion irradiation, and on employing advanced in-situ and quasi-in-situ TEM, APT, synchrotron radiation techniques, and nanomechanical testing to quantitatively decipher the synergistic evolution of irradiation-induced defects, solutes, precipitates, and interfaces. Theoretically, a multi-scale framework that integrates first-principles calculations, MD, rate theory and kinetic Monte Carlo, phase-field modeling, and crystal plasticity simulations should be combined with machine-learning interatomic potentials and data-driven material design strategies to bridge the gap from atomic-scale defect reactions to macroscopic property degradation and lifetime prediction. Beyond their role as surrogate potentials, machine learning methods are increasingly reshaping this research paradigm: they enable high-throughput screening of alloy compositions, accelerate the discovery of irradiation-resistant chemistries, automate the identification and quantification of irradiation-induced defects from microscopy images, and establish quantitative composition–microstructure–property linkages from heterogeneous experimental and simulation datasets. These capabilities not only reduce the reliance on costly neutron irradiation campaigns but also provide a powerful route to extrapolate limited experimental observations into broader design spaces, thereby substantially enhancing the predictive power and efficiency of irradiation damage studies. From the engineering application perspective, irradiation resistance, corrosion resistance, manufacturability (including weldability), cost, and standardization requirements must be holistically balanced to develop high-performance nickel-based alloys and composite structural material systems that possess both long-term microstructural stability under irradiation and economic viability, thereby providing the critical material foundation for the safe, efficient, and long-life operation of advanced nuclear energy systems.

## Figures and Tables

**Figure 1:**
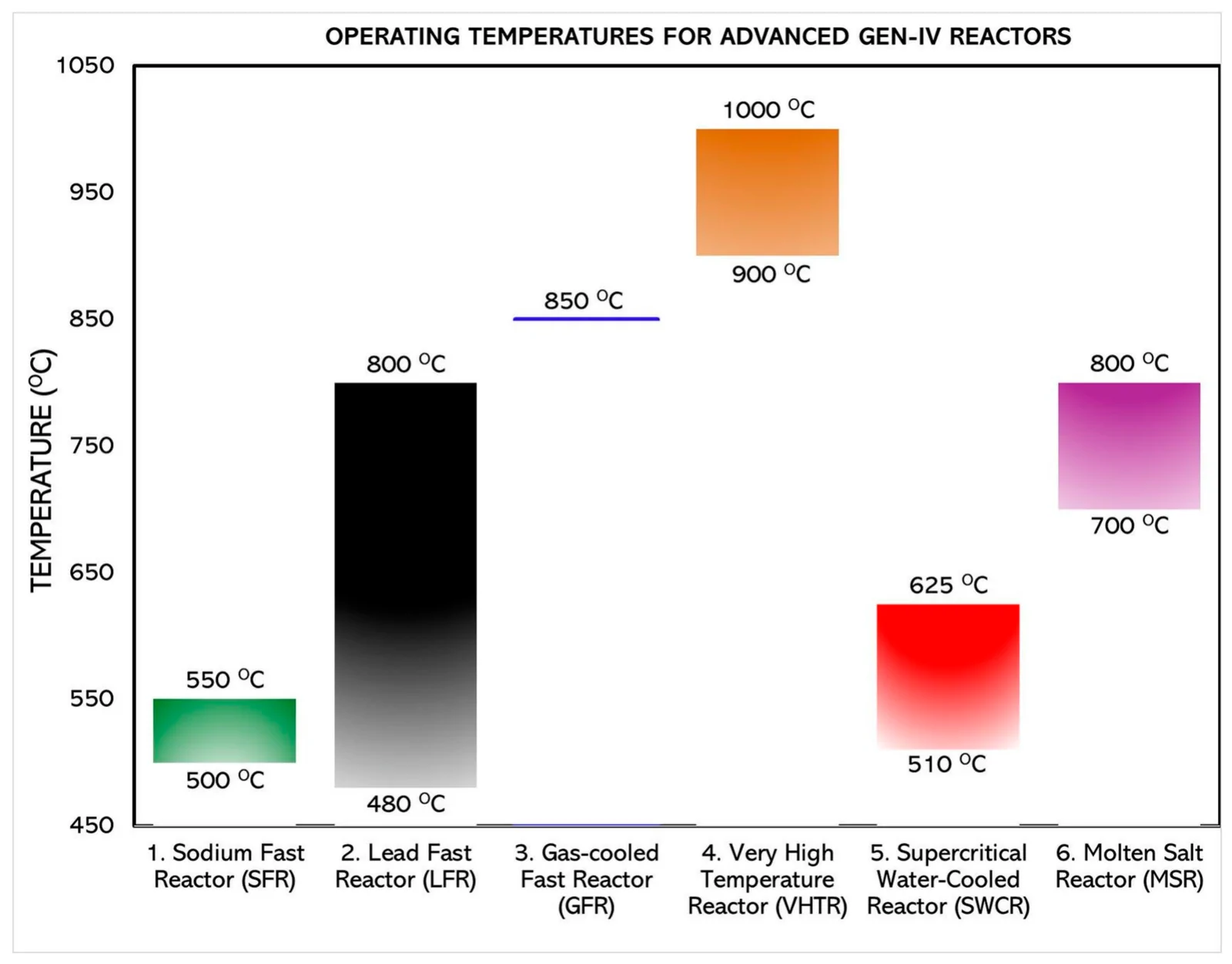
Comparison of the operational regimes characteristic of various Gen-IV reactor designs [2].

**Figure 2:**
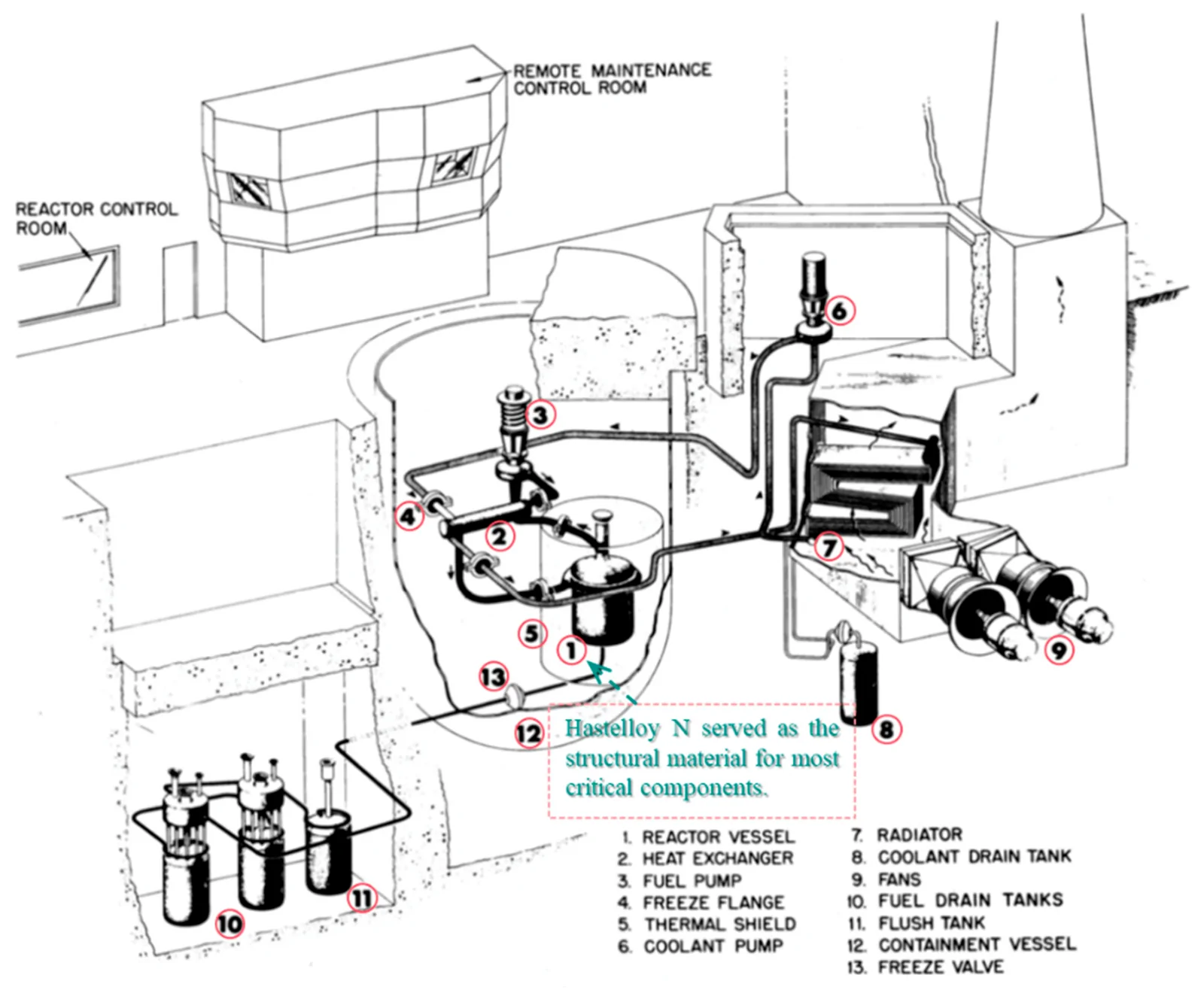
Circuit design of the molten salt experimental reactor and the application of Hastelloy N alloy therein [23,24].

**Figure 3:**
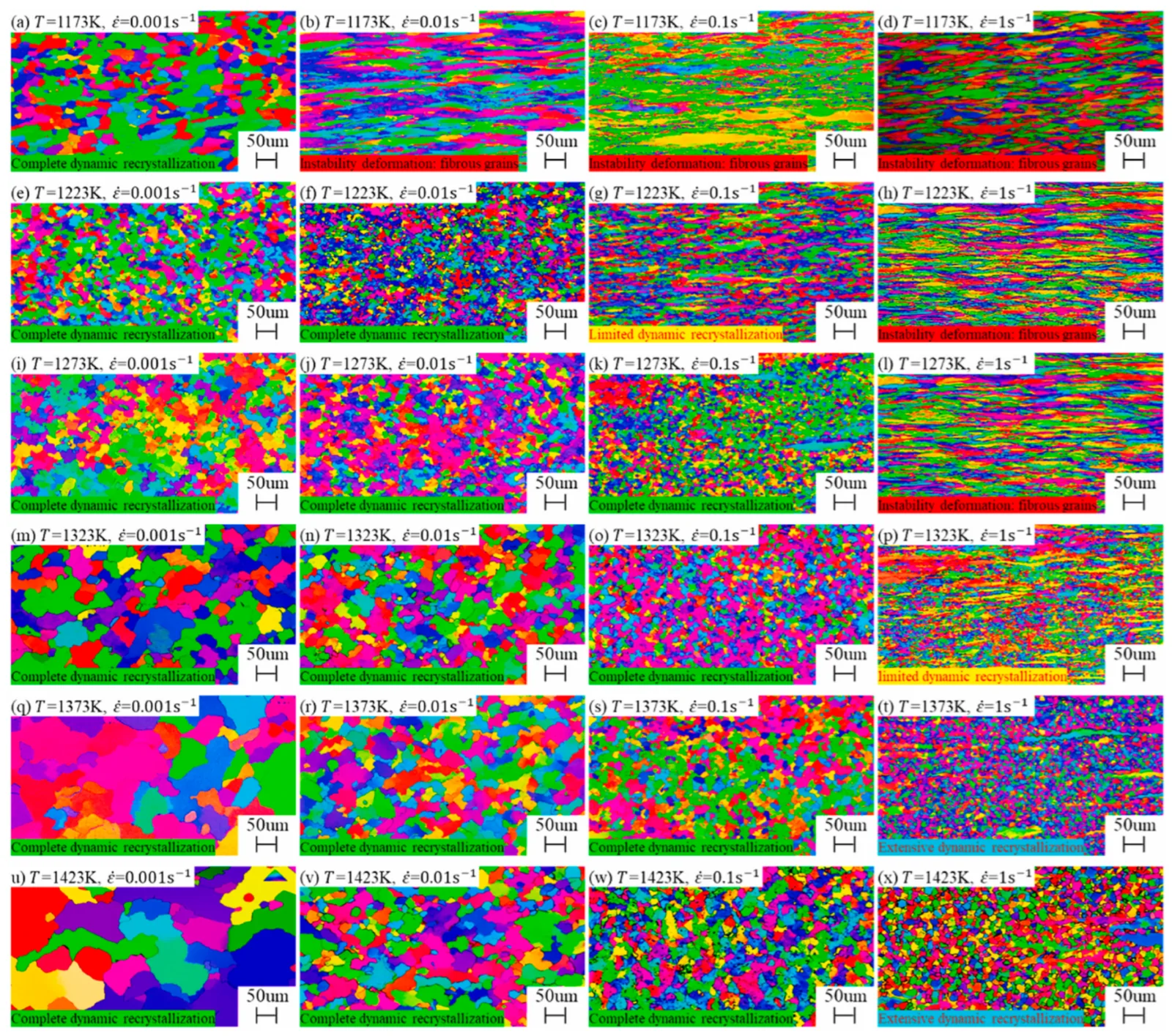
Microstructural response of the GH4706 alloy to hot compression performed across a range of temperatures and strain rates [50].

**Figure 4:**
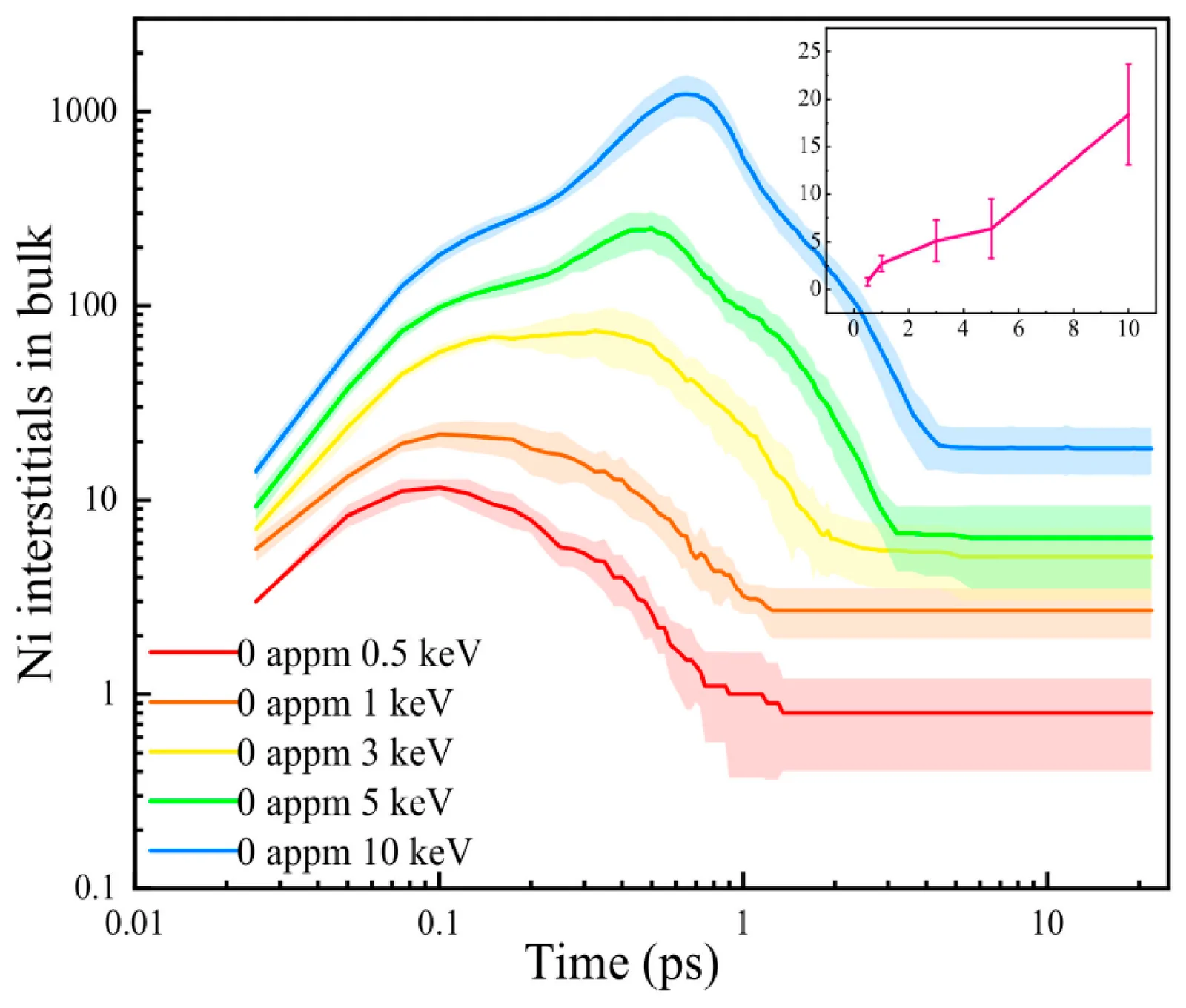
Temporal evolution of the self-interstitial atom population in pure nickel at various PKA energies, with the inset showing the corresponding number of stable residual defects [73].

**Figure 5:**
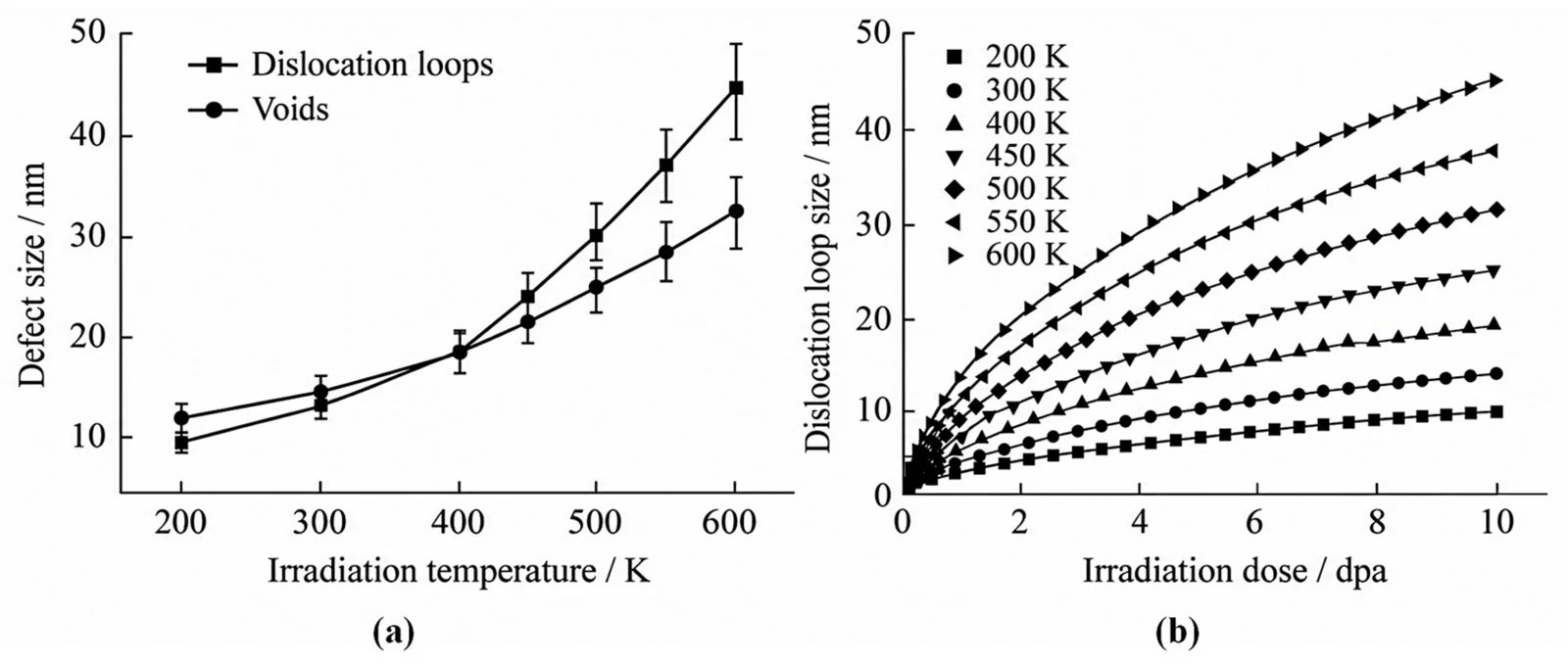
Dependence of defect dimensions on simulation temperature and irradiation dose for Hastelloy C276 alloy: (**a**) sizes of dislocation loops and voids as a function of temperature; (**b**) effect of irradiation temperature and dose on the sizes of dislocation loops [84].

**Figure 6:**
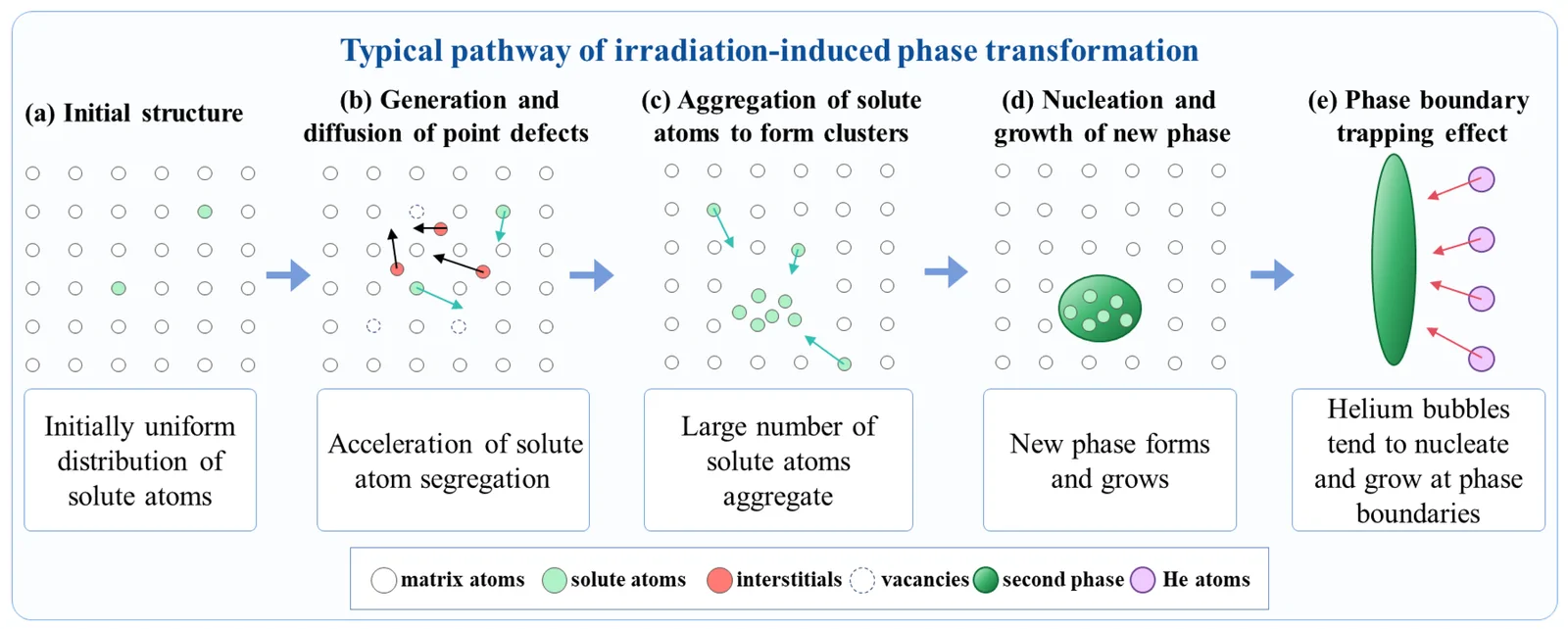
Schematic of the irradiation-induced phase transformation process.

**Figure 7:**
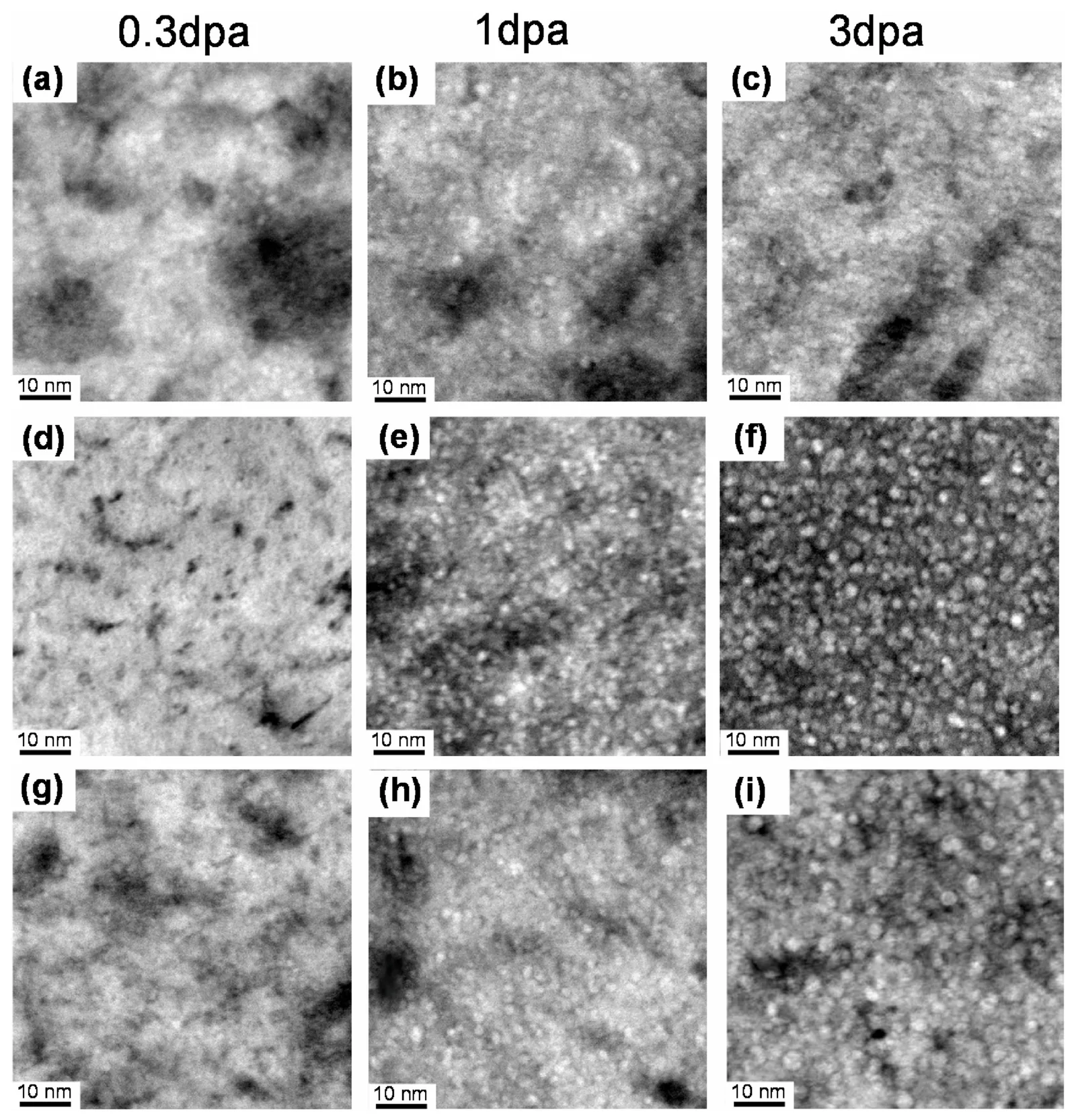
Bright-field images of helium bubbles within the peak helium concentration region after He ion irradiation to different doses: (**a**–**c**) TM-718 alloy; (**d**–**f**) SLM-718 alloy; (**g**–**i**) SLM-718-HT alloy [93].

**Figure 8:**
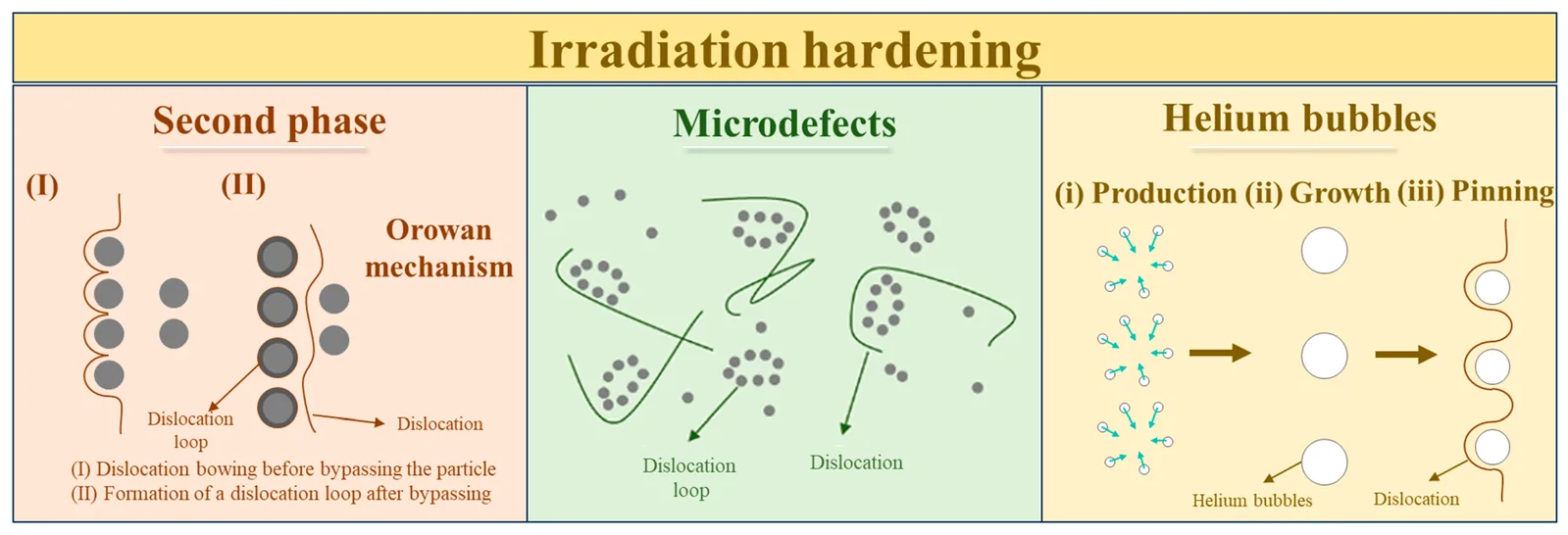
Formation mechanism of irradiation hardening.

**Figure 9:**
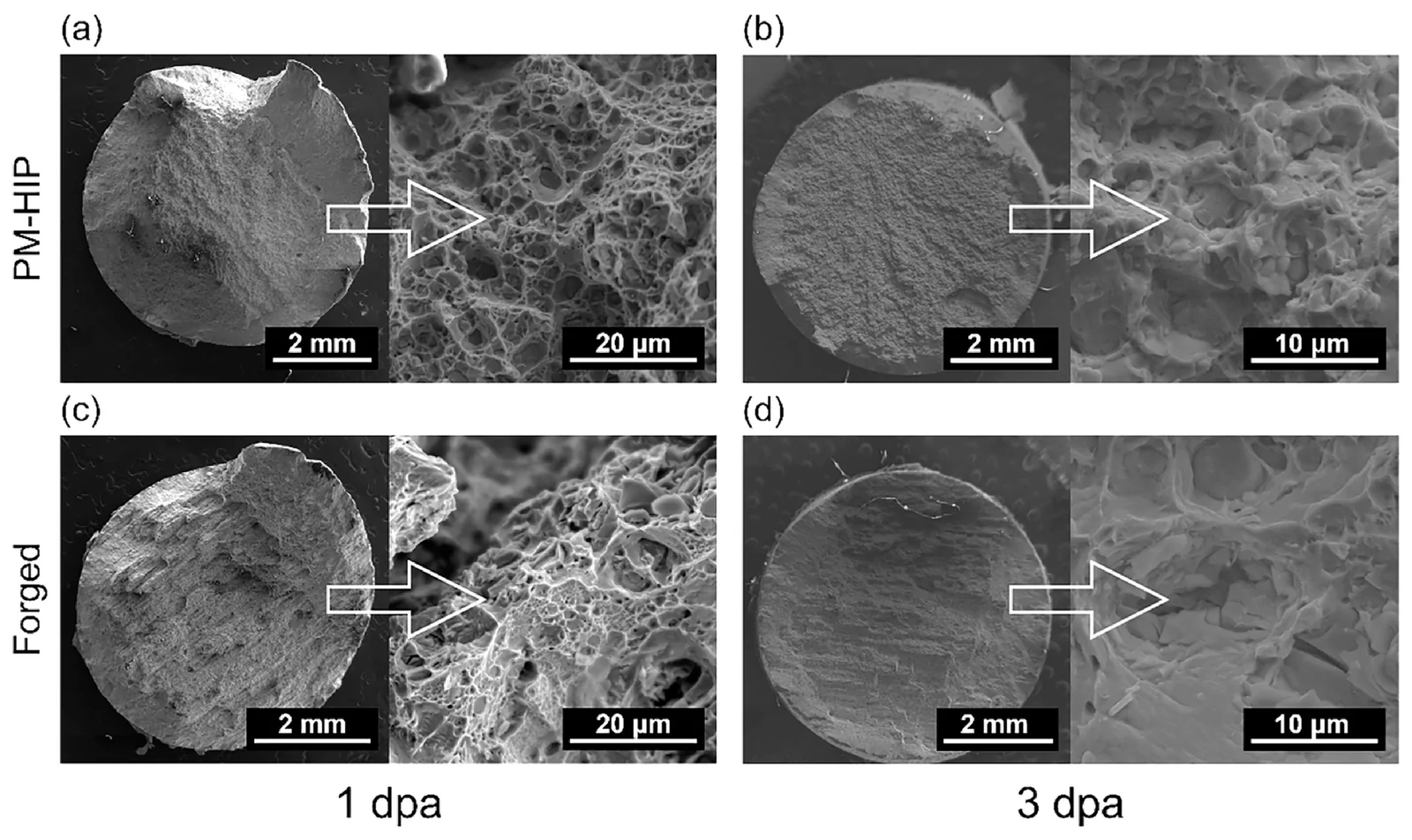
Post-tensile fracture morphologies of PM-HIP Alloy 625 irradiated to (**a**) a target dose of 1 dpa (measured 0.53 dpa) and (**b**) 3 dpa (measured 3.69 dpa). Fractographs for forged Alloy 625 subjected to identical tensile testing after irradiation to (**c**) 1 dpa (measured 0.52 dpa) and (**d**) 3 dpa (measured 3.84 dpa) are also shown [100].

**Figure 10:**
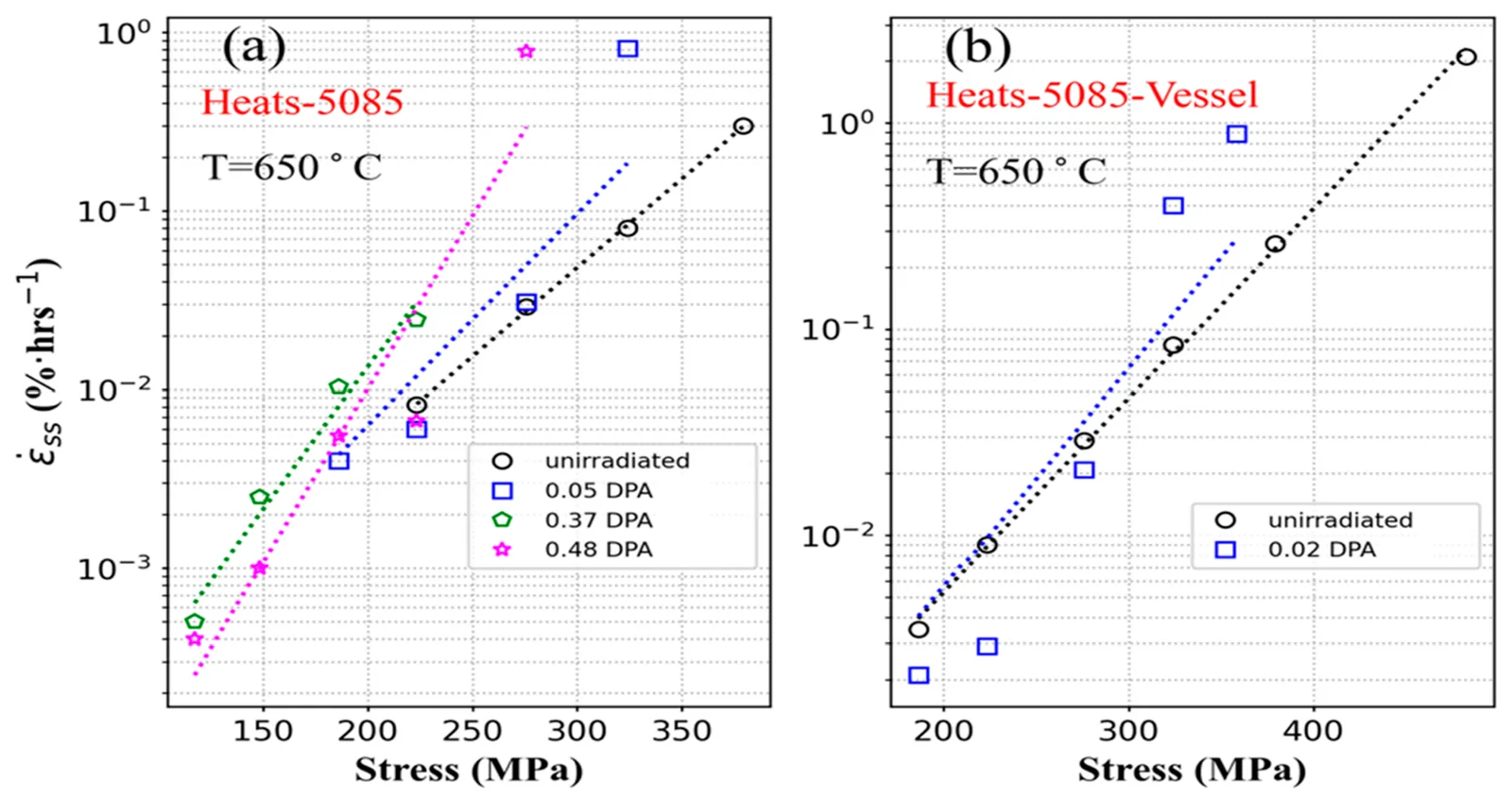
Fitting results of creep rate data for Hastelloy N samples in the MSRE core (neutron irradiation and fluoride molten salt, (**a**) and in its pressure vessel (neutron irradiation and N_2_ with 2–5% O_2_ gas mixture, (**b**) based on the single-atom statistical model (SASM) [105].

**Figure 11:**
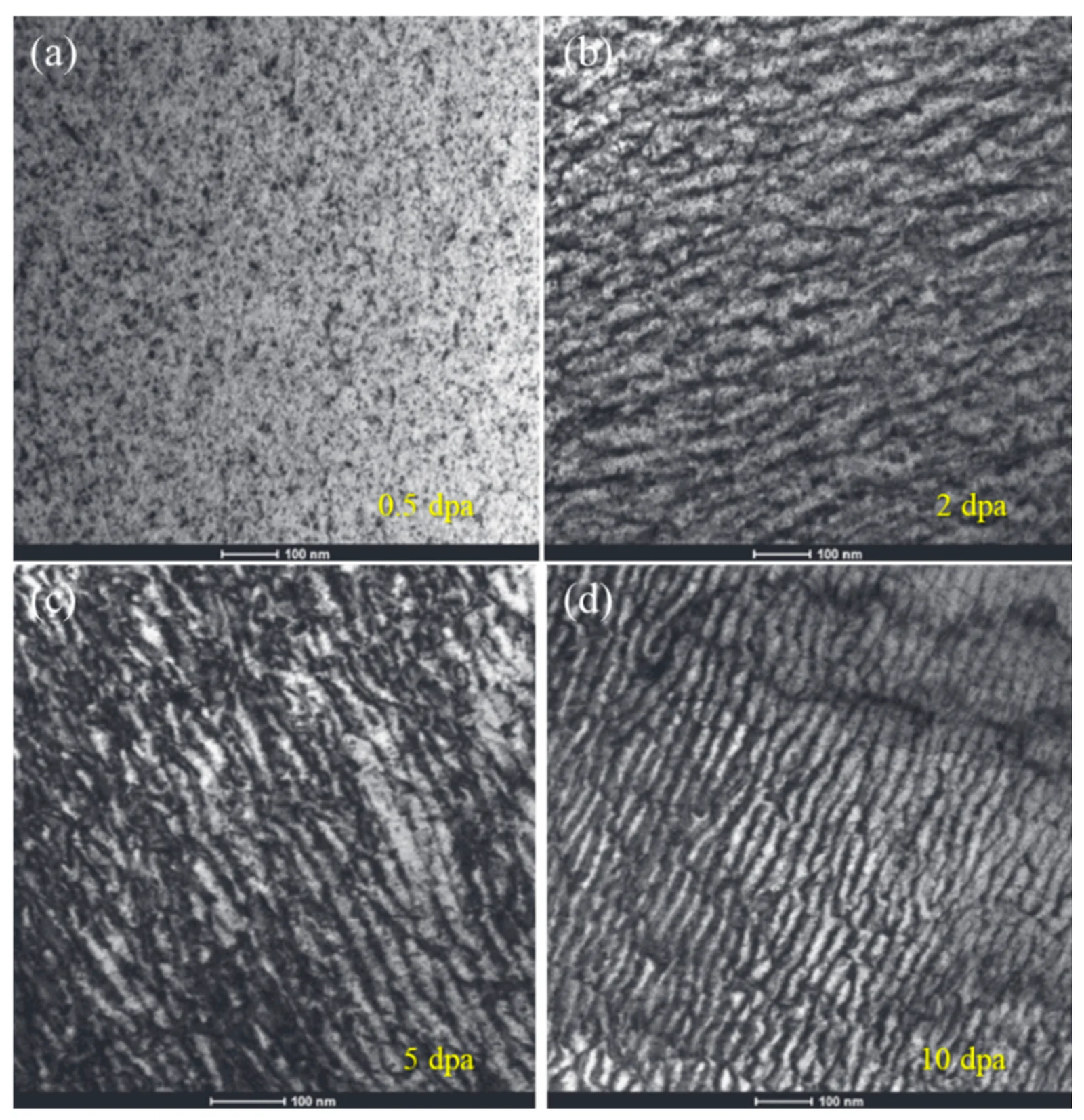
TEM images of Hastelloy N alloy after Xe ion irradiation to different doses: (**a**) 0.5 dpa, (**b**) 2 dpa, (**c**) 5 dpa, and (**d**) 10 dpa [106].

**Figure 12:**
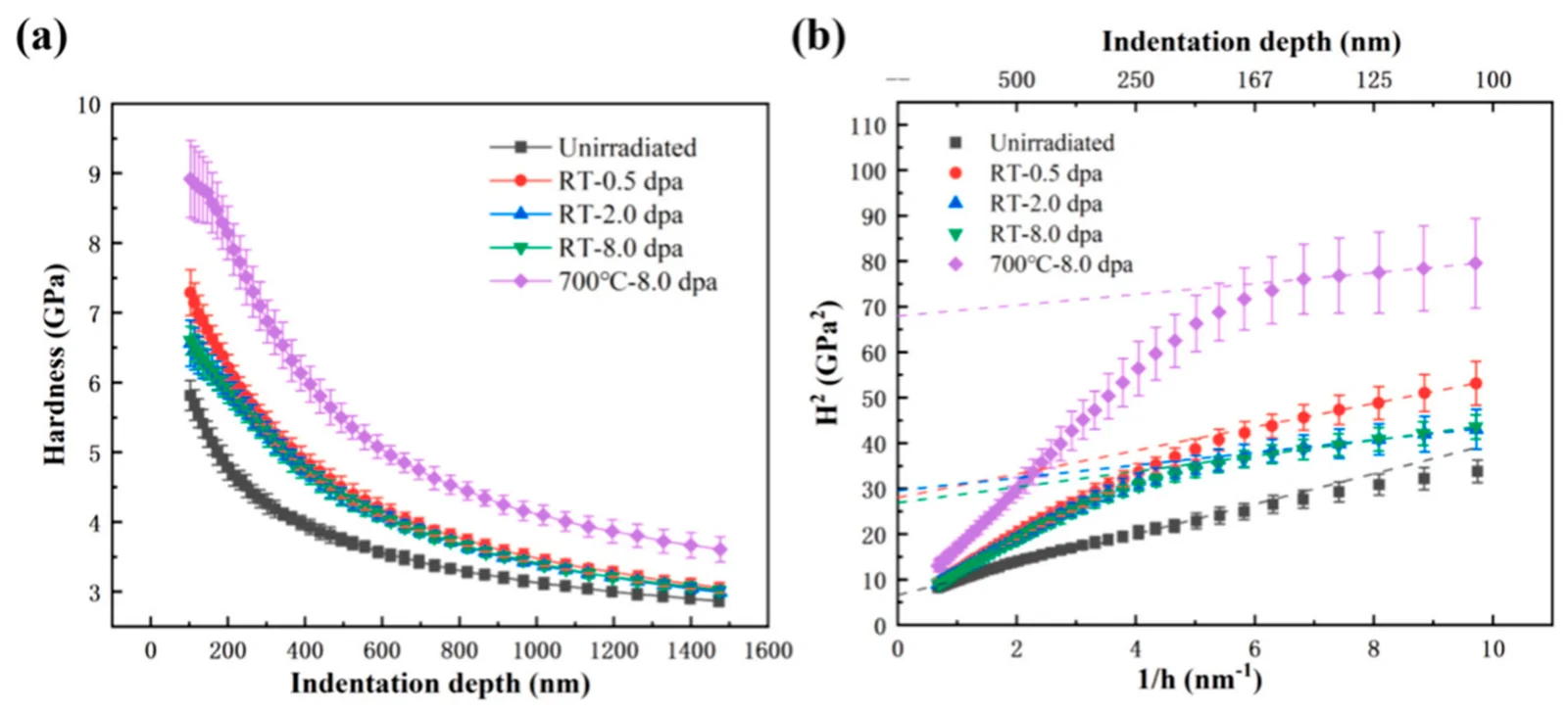
Nanoindentation response of GH3535 alloy for varying irradiation doses and temperatures: (**a**) nanohardness as a function of indentation depth under the tested doses and temperatures, and (**b**) the corresponding H^2^–1/h curves [96].

**Figure 13:**
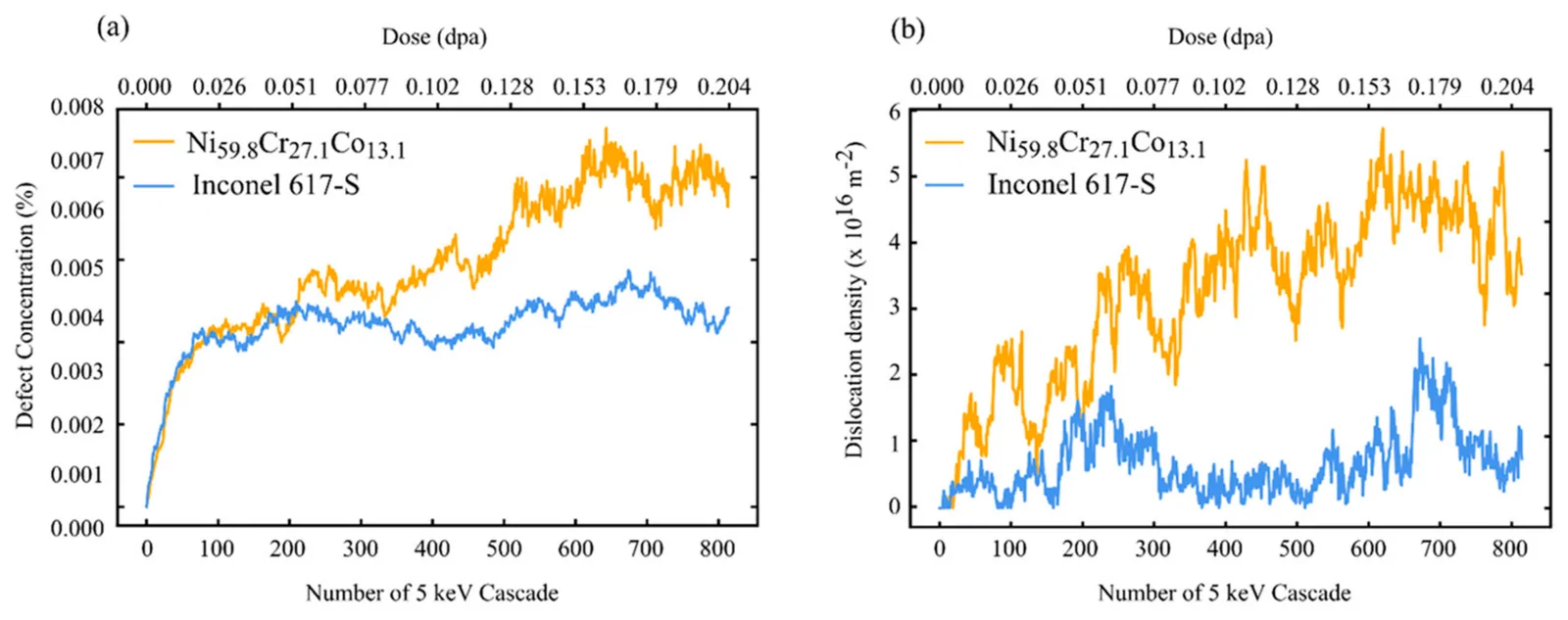
Variation of defect concentration and dislocation density with cascade number (dose) for Inconel 617: (**a**) defect concentration and (**b**) dislocation density [116].

**Figure 14:**
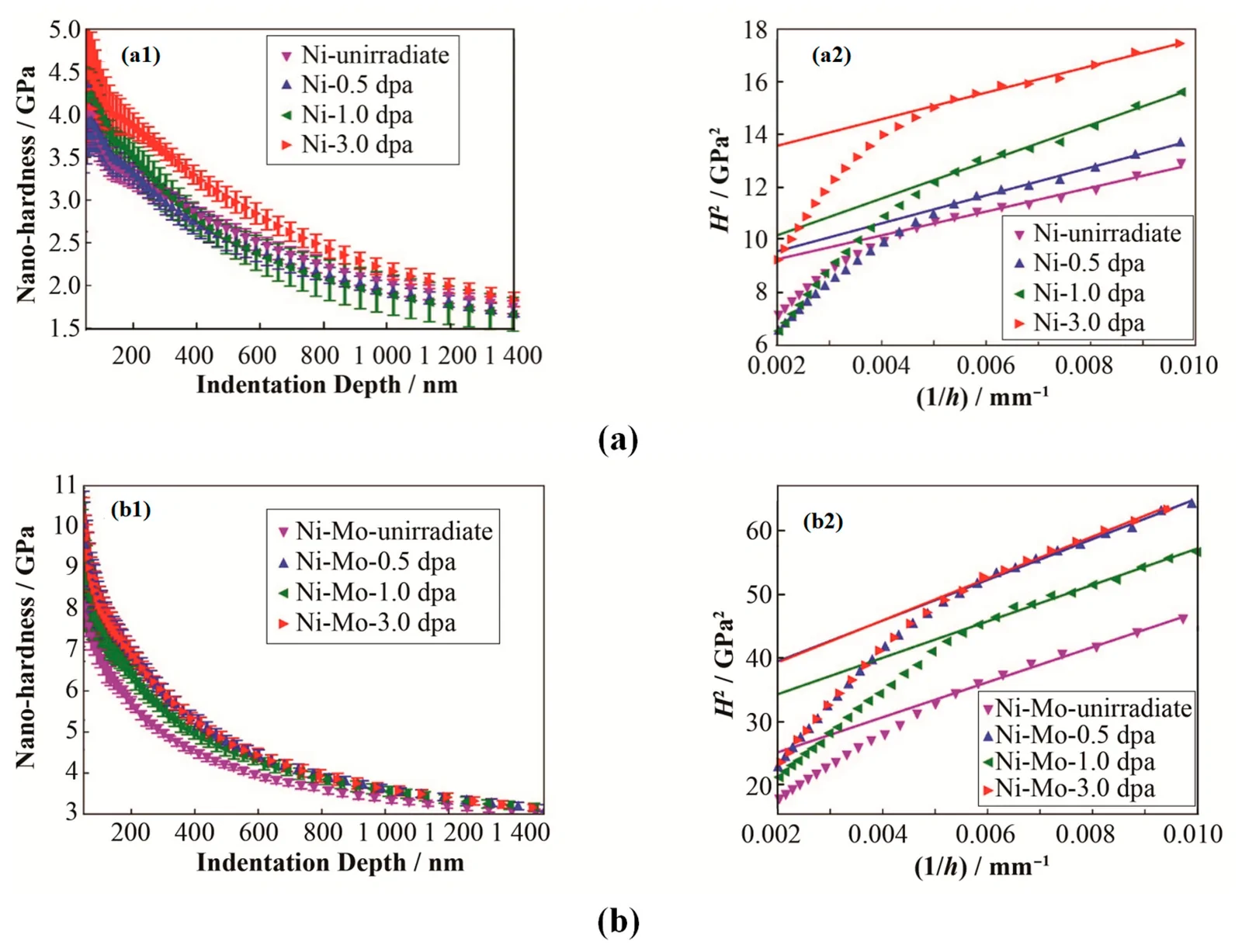
Nanoindentation response of pure Ni and Ni–Mo alloy under different irradiation doses: (**a**) pure Ni, with (**a1**) depth-dependent nano-hardness and (**a2**) H^2^ vs. 1/h plots for average hardness of irradiated and unirradiated samples; (**b**) Ni–Mo alloy, with (**b1**) analogous depth profiles and (**b2**) corresponding H^2^–1/h relationships [132].

**Figure 15:**
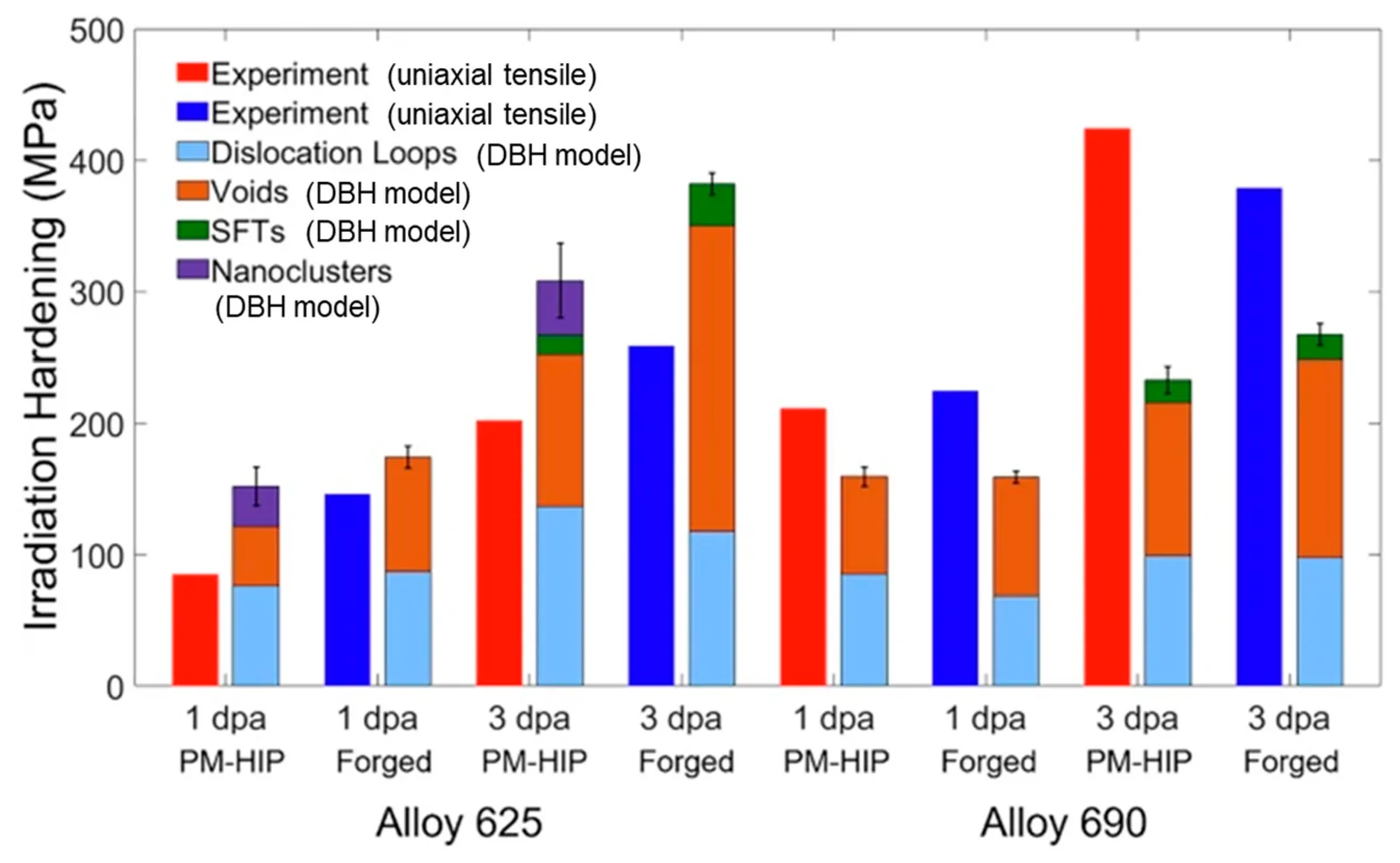
Comparison of experimentally measured irradiation hardening (red bars: PM-HIP Alloy 625 and Alloy 690; blue bars: forged Alloy 625 and Alloy 690) with the corresponding contributions predicted by the dispersed barrier hardening (DBH) model from dislocation loops, voids, SFTs, and nanoclusters at different irradiation doses [100].

**Figure 16:**
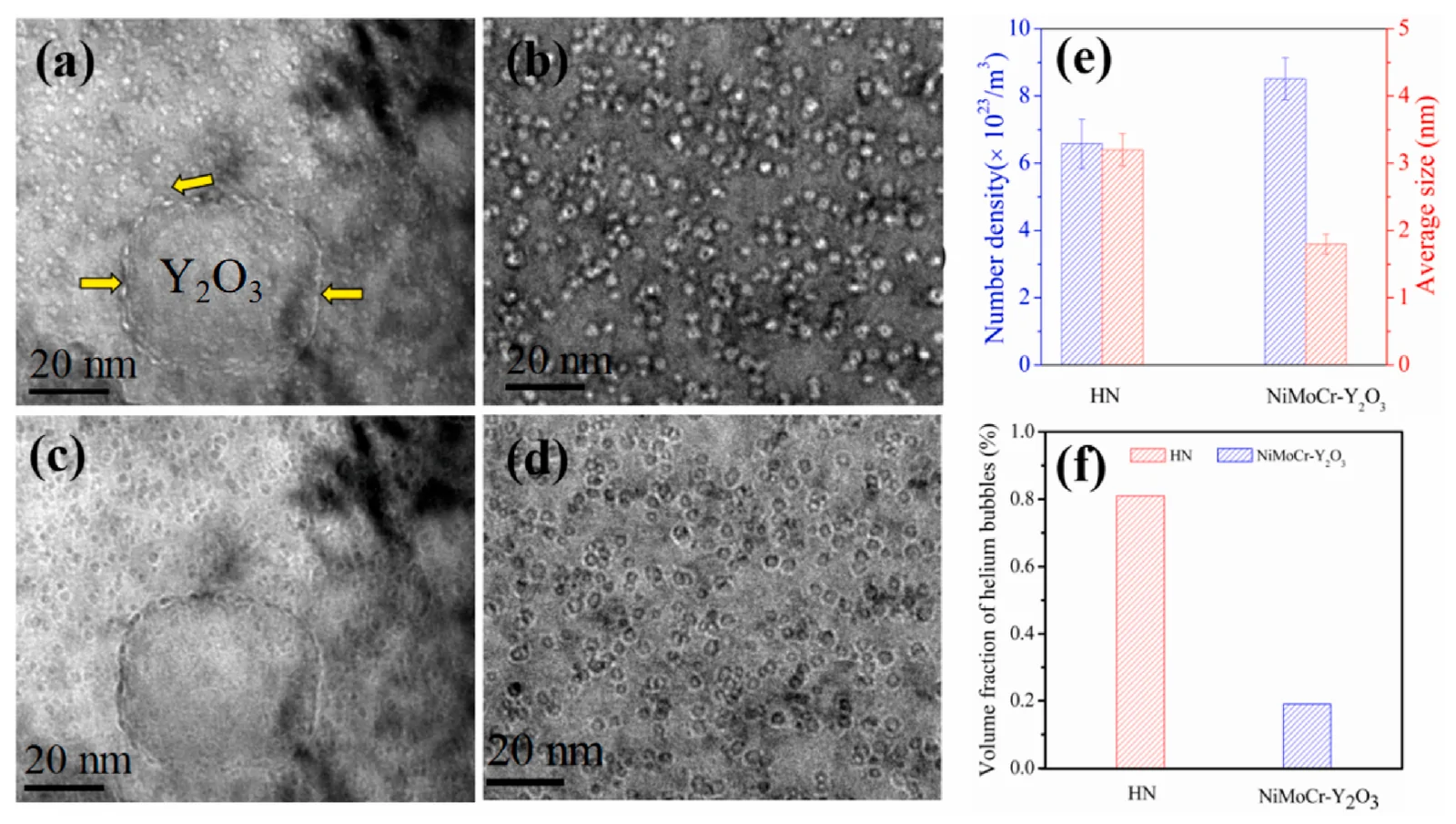
TEM images of helium bubbles at identical peak damage depth are presented: under-focus images for (**a**) NiMoCr–Y_2_O_3_ alloy and (**b**) Hastelloy N alloy, over-focus images for (**c**) NiMoCr–Y_2_O_3_ alloy and (**d**) Hastelloy N alloy, (**e**) the average size, number densities, and (**f**) the volume fraction of helium bubbles in both alloys [142].

**Figure 17:**
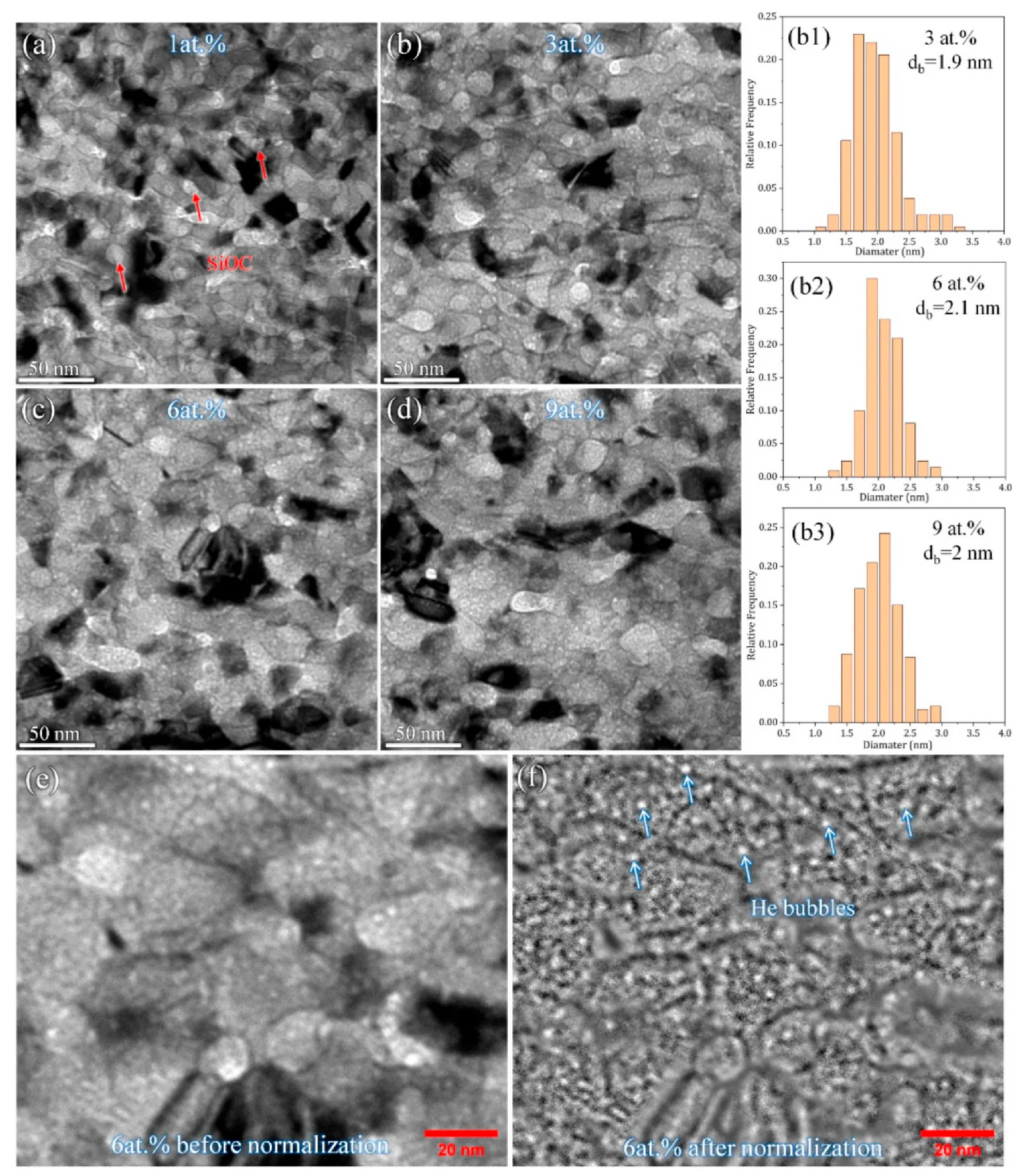
In-situ TEM images (**a**–**f**) of Ni/SiOC samples irradiated with He ions at 300 °C and the corresponding helium bubble size distribution results (**b1**–**b3**) (Note: red arrows indicate the amorphous phase; white arrows indicate helium bubbles) [145].

**Table 1:** Chemical compositions of Hastelloy N alloy [18].

Elements	Ni	Mo	Cr	Fe	Mn	Si	Al	Ti	Co	C
wt.%	Bal.	16.5	6.96	4.2	0.71	0.46	≤0.2		≤0.02	0.05

**Table 2:** Chemical compositions of Inconel 718 alloy [28].

Elements	Ni	Fe	Cr	Nb	Mo	Ti	Co	Al
wt.%	Bal.	19.86	19.09	5.36	2.94	1.10	0.07	0.55

**Table 3:** Chemical compositions of Inconel 617 alloy [33].

Elements	Ni	Cr	Co	Mo	Fe	Al	Ti	C	Si
wt.%	Bal.	20.8	13.1	9.12	0.8	1.28	0.22	0.07	0.11

**Table 4:** Nanoindentation hardness data of GH3535 alloy as a function of irradiation dose and temperature [96].

Ion	Temperature	Dose (dpa)	Nanohardness (GPa)
Fe	RT	0.0	2.57 ± 0.03
		0.5	5.30 ± 0.04
		2.0	5.44 ± 0.01
		8.0	5.19 ± 0.02
	700 °C	8.0	8.24 ± 0.06

## Data Availability

No new data were created or analyzed in this study. Data sharing is not applicable to this article.
